# Natural Genetic Diversity in Tomato Flavor Genes

**DOI:** 10.3389/fpls.2021.642828

**Published:** 2021-06-04

**Authors:** Lara Pereira, Manoj Sapkota, Michael Alonge, Yi Zheng, Youjun Zhang, Hamid Razifard, Nathan K. Taitano, Michael C. Schatz, Alisdair R. Fernie, Ying Wang, Zhangjun Fei, Ana L. Caicedo, Denise M. Tieman, Esther van der Knaap

**Affiliations:** ^1^Center for Applied Genetic Technologies, University of Georgia, Athens, GA, United States; ^2^Institute for Plant Breeding, Genetics and Genomics, University of Georgia, Athens, GA, United States; ^3^Department of Computer Science, Johns Hopkins University, Baltimore, MD, United States; ^4^Boyce Thompson Institute, Ithaca, NY, United States; ^5^Max-Planck-Institut für Molekulare Pflanzenphysiologie, Potsdam, Germany; ^6^Center of Plant Systems Biology and Biotechnology, Plovdiv, Bulgaria; ^7^Department of Biological Sciences, Mississippi State University, Starkville, MS, United States; ^8^U.S. Department of Agriculture, Agricultural Research Service, Robert W. Holley Center for Agriculture and Health, Ithaca, NY, United States; ^9^Biology Department, University of Massachusetts Amherst, Amherst, MA, United States; ^10^Horticultural Sciences, University of Florida, Gainesville, FL, United States; ^11^Department of Horticulture, University of Georgia, Athens, GA, United States

**Keywords:** flavor, tomato, genetic, diversity, metabolomics, breeding

## Abstract

Fruit flavor is defined as the perception of the food by the olfactory and gustatory systems, and is one of the main determinants of fruit quality. Tomato flavor is largely determined by the balance of sugars, acids and volatile compounds. Several genes controlling the levels of these metabolites in tomato fruit have been cloned, including *LIN5*, *ALMT9*, *AAT1*, *CXE1*, and *LoxC*. The aim of this study was to identify any association of these genes with trait variation and to describe the genetic diversity at these loci in the red-fruited tomato clade comprised of the wild ancestor *Solanum pimpinellifolium*, the semi-domesticated species *Solanum lycopersicum cerasiforme* and early domesticated *Solanum lycopersicum*. High genetic diversity was observed at these five loci, including novel haplotypes that could be incorporated into breeding programs to improve fruit quality of modern tomatoes. Using newly available high-quality genome assemblies, we assayed each gene for potential functional causative polymorphisms and resolved a duplication at the *LoxC* locus found in several wild and semi-domesticated accessions which caused lower accumulation of lipid derived volatiles. In addition, we explored gene expression of the five genes in nine phylogenetically diverse tomato accessions. In general, the expression patterns of these genes increased during fruit ripening but diverged between accessions without clear relationship between expression and metabolite levels.

## Introduction

Flavor is defined as the perception of food by multiple senses, including taste and olfaction (Baldwin et al., 2000; Small and Prescott, 2005). Flavor is one of the main determinants of produce quality, especially when consumed as non-processed food. Consumers preferred tomato (*Solanum lycopersicum* var. *lycopersicum*) flavor is determined by the right balance of sugars and organic acids, as well as a range of volatile organic compounds, the latter detected primarily by olfaction (Baldwin et al., 2000; Tandon et al., 2003; Tieman et al., 2012).

Despite the relevance to consumer appeal, produce flavor has been overlooked in breeding programs for decades (Tieman et al., 2017; Klee and Tieman, 2018). Instead, recent crop improvement has focused on agronomic traits, such as yield and disease resistance, which are important to growers and producers. This selection process has led to less flavorful modern cultivars in a range of crops, and in particular to a high level of consumer dissatisfaction of tomato (Tieman et al., 2017). An appropriate balance of sugars and organic acids as well as a rich and diverse volatile profile must be achieved to improve modern varieties that are considered less flavorful than heirlooms. Unlike sugars and acids, most volatiles are active at picomolar to nanomolar concentrations, which would permit flavor improvement without compromising yield. However, metabolite quantification can be technically challenging, labor-intensive and expensive, especially for breeding programs. Thus genetic improvement using molecular selection for alleles of known genes that enhance fruit flavor is one of the major goals in current breeding programs (Rambla et al., 2014; Tieman et al., 2017).

More than 400 volatiles have been detected in tomato (Buttery et al., 1989). Empirical studies, including extensive biochemical characterization and trained consumer panels, have shown that only 20 to 30 volatiles are correlated to consumer liking (Tandon et al., 2003; Tieman et al., 2012). Different volatiles contribute to several aspects of flavor. For example, lipid-derived volatiles, such as *Z*-3-hexen-1-ol and hexyl alcohol, are associated with tomato flavor intensity (Li et al., 2020). Acetate esters such as isobutyl acetate and 2-methylbutyl acetate confer a floral-like or fruity aroma and are negatively associated with good tomato flavor (Goulet et al., 2012).

The major biochemical pathways involved in metabolite production and accumulation in tomato have been partially elucidated in recent years (Klee and Tieman, 2018; Martina et al., 2021). The key underlying genes in these pathways were often identified using introgression lines, relying on interspecific variation between cultivated tomato and the distantly related green-fruited *Solanum pennellii* (Fridman et al., 2004; Goulet et al., 2012, 2015). The high rate of divergence between the parents facilitated the identification of the genes by functional or positional cloning approaches. However, the likely nucleotide polymorphisms leading to trait evolution resulting from domestication remains unknown for most known flavor genes.

Genetic variation within cultivated tomato and the closely related red-fruited wild relatives has been explored through genome-wide association studies (GWAS). These studies have identified hundreds of loci involved in the production of multiple compounds, which paved the way for a targeted molecular breeding approach to recover the flavor in modern tomatoes (Tieman et al., 2017; Zhu et al., 2018; Zhao et al., 2019; Razifard et al., 2020). Several significant GWAS loci colocalize with known genes, demonstrating that in many cases these same genes that were identified among distantly related species underlie the accumulation of metabolites in the red-fruited tomato clade as well. For example, using new long-read sequencing technology, the natural diversity at the *Non-Smoky Glycosyl Transferase* gene, known to control the emission of guaiacol and methylsalicylate via sugar conjugation, showed multiple haplotypes that were associated with the levels of these volatiles (Tikunov et al., 2013; Alonge et al., 2020). Specifically, structural variants (SVs) consisting of deletions, insertions, duplications, inversions and translocations of a certain size, usually above 50-100 bp (Torkamaneh et al., 2018) have often been found to underlie phenotypic variation in tomato (Xiao et al., 2008; Mu et al., 2017; Soyk et al., 2017; Wu et al., 2018; Alonge et al., 2020).

Flavor is a key trait in the domestication syndrome of fruit crops (Meyer and Purugganan, 2013). The flavor palette of tomato changed dramatically during the domestication and diversification of the species (Schauer et al., 2006; Rambla et al., 2017; Zhu et al., 2018). The fully wild, red-fruited species *Solanum pimpinellifolium* (SP) gave rise to *Solanum lycopersicum* var. *cerasiforme* (SLC) in South America from which cultivated tomato *Solanum lycopersicum* var. *lycopersicum* (SLL) eventually arose in Mexico (Razifard et al., 2020). As an intermediate between SLL and SP, SLC accessions have been shown to have high genetic and phenotypic diversity. The goal of this study was to investigate the genetic diversity and gene expression in a set of five genes associated with fruit flavor and to identify beneficial haplotypes that could be incorporated into breeding germplasm. To accomplish this aim, we used a genetically well characterized collection of SP, SLC and SLL from South and Central America (collectively called the Varitome collection) and a combination of whole-genome and RNA sequences, as well as their metabolic profiles.

## Materials and Methods

### Plant Material

The Varitome collection consists of 166 accessions from South and Central America (Mata-Nicolás et al., 2020). Using whole genome sequencing and passport information, the accessions are classified into SP, SLC, and SLL (Razifard et al., 2020). Each phylogenetic group was divided in several subpopulations: three SP subpopulations with well-defined geographical origin (South Ecuador, SP-SECU; Northern Ecuador, SP-NECU; and Peru, SP-PER); five SLC subpopulations, three from South America (Ecuador, SLC-ECU; Peru, SLC-PER; and the San Martin region of Peru, SLC-SM), one with wide geographical distribution in Central, Northern South and Southern North America (collectively called SLC-CA) and one from Mexico (SLC-MEX). The SLL represented one subpopulation of early domesticated landraces from Mexico (Razifard et al., 2020). Eight accessions were excluded from the haplotype analysis because they were classified as SLC admixtures or lacked the metabolic profiles. The plants were grown in the fields at the University of Florida, North Florida Research and Education Center–Suwannee Valley in the spring of 2016 using standard commercial production practices. The plants used for transcriptomic analysis were grown in the greenhouse at the Ohio State University, Columbus, OH, United States at 20°C night and 30°C day temperature, and a 16/8 hr light/dark cycle. Seedlings were transplanted in 1.6-gallon pots in Sungrow Metro soil mix supplemented with three tablespoons of a 5:1 blend of Florikan Nutricote Total 18-6-8 270day and Florikan Meg-Iron V Micronutrient Mix. The plants were hand watered when the pots were dry but before wilting.

### Variant Calling

Raw ILLUMINA read files of the Varitome accessions were downloaded from NCBI (^1^ SRA: SRP150040, BioProject: PRJNA454805). The read quality of raw sequencing data was evaluated using FastQC^2^. Low quality reads (read length less than 20) and adapter sequences were trimmed with the tool Trimmomatic (Bolger et al., 2014a). The reads were then aligned to SL4.0 build of tomato reference genome^3^ using “speedseq align” component of SpeedSeq framework (Chiang et al., 2015).

SNP and small INDEL variant calling was performed using GATK v3.8 following GATK best practices workflow (Van der Auwera et al., 2013). HaplotypeCaller was used to produce individual gVCF files, which were later combined in a multi-sample VCF file with GenotypeGVCFs. SNPs and INDELs were extracted using SelectVariants. Raw SNPs were then filtered based on the following quality parameters: MQ > 40, QD > 2, FS < 60, MQRankSum > -12.5 and ReadPosRankSum > -8. Similarly, raw INDELs were filtered using QD > 2, FS > 200, ReadPosRankSum < -20. Variants with missing data in more than 10% of the accessions were filtered out.

SVs (> 100 bp) were detected using aligned BAM files and its corresponding splitter and discordant files using “lumpyexpress” function of LUMPY (Layer et al., 2014). The resulting SVs were filtered based on following criteria: minimum number of pair end (PE) 1, minimum number of split read (SR) 1, SR less than or equal to PE, and total number of supporting reads greater than or equal to half of average read depth and less than or equal to three times of average read depth. Then, filtered SVs were merged to generate a single multi-sample VCF file using SURVIVOR (Jeffares et al., 2017). SVs within a maximum allowed distance of 500 bp were merged.

The same pipeline was employed to analyze a subset of cultivated accessions representative of the genetic diversity within heirloom and modern varieties, previously sequenced (Tieman et al., 2017). The sequencing data were downloaded from NCBI (SRA: SRP045767, SRP094624, PRJNA353161), and only accessions with a coverage larger than 5x were used. All the filtering parameters were identical except the missing data cutoff. In this case, variants with missing data in more than 50% of the accessions were filtered out as a result of the lower sequencing coverage in the Tieman et al. (2017) data compared to the Varitome data.

### Association Mapping

First, we compiled a list of known genes affecting fruit flavor (Table 1). To our knowledge, the list included all the known genes affecting sugars, acids, acetate esters, lipid-derived volatiles, phenylalanine-derived volatiles, guaiacol, methylsalicylate and carotenoids. Variant data (SNPs, INDELs and SVs) of the loci described in Table 1 as well as 1 Mb upstream and 1 Mb downstream of the transcription start and termination were extracted from the multi-sample VCF files using bedtools (Quinlan and Hall, 2010), and used for the local association analysis. The ITAG4.1 version of the annotation was used to delimit gene coordinates. Phenotypes deviating from normality (*p*-Value from Shapiro test < 0.01) were normalized using quantile normalization. Genome-wide kinship matrix was calculated based on SNPs using the Centered IBS method, to generate the Hapmap files in TASSEL 5.2.44 (Bradbury et al., 2007). Associations between the genotype and phenotype were estimated using BLINK (Huang et al., 2019) model in GAPIT (version 3) (Tang et al., 2016). Minor allele frequency was set to 2% for the analysis. This was set lower than the usual 5% threshold to account for rare alleles in the collection which we did not want to exclude as they could have an impact on protein function. The significance thresholds for the association were set to a –logP of >6.59 and 4.11 representing *p*-Values of 0.01 and 0.05 respectively, after multiple testing correction by the Benjamini and Hochberg FDR estimation. The phenotypic variation explained (PVE) by a SNP was approximated subtracting the likelihood ratio-based R^2^ of the model with the SNP and the likelihood ratio-based R^2^ of the model without the SNP (Xu et al., 2016).

**TABLE 1:** Compilation of known flavor-related genes in tomato.

**Metabolites**	**Gene**	**Gene ID**	**Genomic position**	**References**
Sugars	*LIN5*	*Solyc09g010080*	SL4.0ch09:3508156-3512282	Fridman et al., 2004

Organic acids (malate)	*ALMT9*	*Solyc06g072920*	SL4.0ch06:42612816-42619107	Ye et al., 2017

Acetate esters	*AAT1*	*Solyc08g005770*	SL4.0ch08:617070-619717	Goulet et al., 2015
	*CXE1*	*Solyc01g108585*	SL4.0ch01:88169038-88170233	Goulet et al., 2012

Lipid-derived volatiles	*LoxC*	*Solyc01g006540*	SL4.0ch01:1119976-1130114	Shen et al., 2014
	*HPL*	*Solyc07g049690*	SL4.0ch07:59963576-59970053	Shen et al., 2014
	*ADH2*	*Solyc06g059740*	SL4.0ch06:35287450..35289927	Speirs et al., 1998
	*LIP1*	*Solyc12g055730*	SL4.0ch12:61316763..61320764	Garbowicz et al., 2018
	*LIP8*	*Solyc09g091050*	SL4.0ch09:66484639-66495126	Li et al., 2020

Phenylalanine-derived volatiles	*PAR1*	*Solyc01g008530*	SL4.0ch01:2578092..2584487	Tieman et al., 2007
	*PAR2*	*Solyc01g008550*	SL4.0ch01:2593768..2597462	
	*AADC2*	*Solyc08g006740*	SL4.0ch08:1306822..1309453	Tieman et al., 2006b
	*AADC2*	*Solyc08g006750*	SL4.0ch08:1332553..1336469	
	*AADC1C*	*Solyc08g068600*	SL4.0ch08:55827604..55829855	
	*AADC1B*	*Solyc08g068610*	SL4.0ch08:55836822..55838978	
	*AADC1D*	*Solyc08g068630*	SL4.0ch08:55860361..55862523	
	*AADC1A*	*Solyc08g068680*	SL4.0ch08:55909433..55911654	
	*PPEAT*	*Solyc02g079490*	SL4.0ch02:42004857-42007233	Domínguez et al., 2020
	*FLORAL4*	*Solyc04g063350*	SL4.0ch04:54805156-54812314	Tikunov et al., 2020

Guaiacol and methylsalicylate	*SAMT*	*Solyc09g091550*	SL4.0ch09:66901227..66903818	Tieman et al., 2010
	*COMT*	*Solyc10g005060*	SL4.0ch10:64725323..64728276	Mageroy et al., 2012

Carotenoids and apocarotenoid volatiles	*PSY1*	*Solyc03g031860*	SL4.0ch03:4234654-4238638	Fray and Grierson, 1993
	*CrtISO*	*Solyc10g081650*	SL4.0ch10:61789271..61794607	Isaacson et al., 2002
	*CYCB*	*Solyc06g074240*	SL4.0ch06:43562526-43564022	Ronen et al., 2000
	*CrtL-e*	*Solyc12g008980*	SL4.0ch12:2334383..2339689	Ronen et al., 1999
	*SlCCD1A*	*Solyc01g087250*	SL4.0ch01:74432005-74442676	Simkin et al., 2004
	*SlCCD1B*	*Solyc01g087260*	SL4.0ch01:74444645-74454599	

Linkage disequilibrium (LD) heatmaps were generated using LDBlockShow 1.33 (Dong et al., 2020) using mean *r*^2^ values. SNPs 1 Mb upstream and downstream of the gene locus were used for LD analyses. Because of high computational demand of the analysis, we used a reduced input data file with one SNP per kb. The reduced data file was generated using “–thin 1000” parameter in VCFtools (Danecek et al., 2011). The results are representative since recombination within the 1-kb window in tomato is insignificant.

### Haplotype Analysis

SNPs and small INDELs within the gene sequence as well as 3 kb upstream of the start site and 1 kb downstream of the termination site were extracted using VCFtools (Danecek et al., 2011). This region was much shorter than the region used for the association mapping because of the unwieldy number of polymorphisms in a larger region as well as the chance of recombination that could result in a large number of haplotypes. SVs detected by Lumpy were not included in the haplotype analysis because of low incidence. Relevant SVs are mentioned in the results section. Additional filter parameters were –mac 4 –max-missing 0.9 –minQ 100. Multiallelic variants were split into multiple rows and left-aligned using BCFTools norm (Li, 2011). Variants were annotated using SnpEff (Cingolani et al., 2012) using a local built database for the SL4.0 tomato reference genome. Since *CXE1* was absent in the ITAG4.1 gene model^4^, we used the FGENESH (Salamov and Solovyev, 2000) tool to predict the gene model and analyzed the locus manually.

The haplotype heatmap was generated using the R package “pheatmap” (Kolde, 2019). The function pheatmap was implemented using the clustering method “ward.D” for accessions (rows) and no clustering method for variants (columns). The number of clusters was set to 6 after testing multiple values, as this value produced the optimal interpretable haplotype clusters at all the analyzed genes. The phylogeny of the accession was extracted from previous whole genome analysis of the same dataset (Razifard et al., 2020). The metabolite content of each accession was classified as low, medium or high depending on the decile position from low: 1st to 5th decile; medium: 6th to 8th decile; high 9th to 10th decile. The variants were classified by their location and functional annotation; variants predicted to affect splicing sites were considered frameshift mutations.

The multiple mean comparison to test significant differences between clusters was conducted in R using a linear model. We used the functions lsmeans from package “emmeans” (Lenth, 2020) to calculate the *p*-Value of pairwise comparisons among clusters and cld from package “multcompView” (Graves et al., 2015) to display the Tukey test, fixing the significance threshold at 0.05.

To generate the haplotype networks, we only used the coding sequence of each gene. A FASTA sequence for each accession and gene was generated by substituting the alternate allele of SNPs and INDELs in the reference sequence using FastaAlternateReferenceMaker from GATK (McKenna et al., 2010). Only the homozygous alternate genotypes were substituted, while the heterozygous genotypes were kept as reference. These were aligned using MAFFT algorithm (Katoh and Standley, 2013) to select the coding sequences according to the ITAG4.1 annotation for each gene. The haplotype networks were constructed using PopART (Leigh and Bryant, 2015) and the minimum spanning tree method (Epsilon = 0) (Bandelt et al., 1999). Sequence from one accession of *S. pennelli* (Bolger et al., 2014b) was included to provide a root for the network.

### Diversity Analysis

Nucleotide diversity (π) was estimated per subpopulation using exclusively SNPs within each gene and flanking sequences (3 kb upstream and 1 kb downstream). The quality thresholds were the same as described before (see “Variant calling”). We classified the SNPs as non-synonymous (resulting in protein changes), synonymous (silent mutations in coding sequence), and non-coding (within introns, UTRs and flanking sequence) by following SnpEff annotation (Cingolani et al., 2012). Then we calculated π estimates per subpopulation using VCFTools (Danecek et al., 2011) using –window-pi function (window of 1000 bp) for non-synonymous, synonymous, non-coding and all SNPs.

### Identification and Genotyping of *LoxC* Duplication

To evaluate whether *LoxC* was duplicated in SP accessions, we used the new high-quality assembly of PAS014479, a SP-PER accession from the Varitome collection that carries the two paralogs (Alonge et al., 2020). The trimmed reads from the Varitome accessions as well as Heinz (SRA: SRP010718) and LA2093 (SRA: SRP267721) were then mapped to the PAS014479_MAS1.0^5^ using the same workflow as described above for the other genes using the SL4.0 reference genome. We aligned *LoxC* and the flanking regions (±50 kb) of PAS014479 to itself and generated a dot-plot to identify identical sequence matches using MUMmer (Kurtz et al., 2004). To check whether the duplication was predicted to be a functional protein, we estimated the gene model using FGENESH web tool and aligned the protein sequences. In addition, we analyzed the alignment files using PAS014479_MAS1.0 as reference genome at *LoxC* locus for a subset of representative accessions using the package “Gviz” (Hahne and Ivanek, 2016). The coordinates of the gene model of the second copy of *LoxC*, denominated *LoxC-SP*, were plotted along with *LoxC* ITAG4.1 gene model.

To genotype the duplication across the Varitome collection *in silico*, we used three approaches: normalized coverage, heterozygosity when aligning to Heinz SL4.0 reference genome, and presence of a deletion when aligning to PAS014479_MAS1.0. At least two out of these three criteria must be met to consider a certain accession to carry *LoxC-SP* featuring both paralogs.

### Metabolic Phenotyping

Fresh fruit volatiles were collected and quantitated as described previously (Tieman et al., 2006a). Sugars and acids were quantitated as described in Vogel et al. (2010).

### Total RNA Isolation, Library Construction, and Sequencing

The tomato maturation timeline for nine accessions was determined prior to collecting the fruit development samples. Five developmental stages per accession were sampled: flower at anthesis, young fruit, mature green fruit, fruit at breaker stage and red ripe fruit and each sample included three biological replicates. Total RNA was isolated using the RNAzol^*RT*^ reagent (Sigma-Aldrich, St. Louis, MO, United States). Strand-specific RNA-Seq libraries were constructed using an established protocol (Zhong et al., 2011). All libraries were quality checked using the Bioanalyzer and sequenced on an Illumina HiSeq 2500 system at Weill Cornell Medicine, NY, United States.

### RNA-Seq Read Processing, Transcript Assembly, and Quantification of Expression

Single-end RNA-Seq reads were processed to remove adapters as well as low-quality bases using Trimmomatic (Bolger et al., 2014a), and trimmed reads shorter than 80 bp were discarded. The remaining reads were subjected to rRNA sequence removal by aligning to an rRNA database (Quast et al., 2013) using Bowtie (Langmead et al., 2009) allowing up to three mismatches. The resulting reads were aligned to the tomato reference genomes (Build SL4.0 see text footnote 3) using STAR (Dobin et al., 2013) allowing up to two mismatches. The gene expression was measured by counting the number of reads mapped to gene regions. Then the gene expression was normalized to the number of reads per kilobase of exon per million mapped reads (RPKM) based on all mapped reads. A principal component analysis was performed for each developmental stage using DESeq2 (Love et al., 2014). Thirteen biological replicates that deviated in the principal component analysis were excluded from the analysis. After this quality filtering, out of the total 45 samples, 36 samples included three biological replicates, seven samples included two biological replicates and two samples were completely excluded. Given the presence of two variables (i.e., genotypes and developmental stages), we used linear modeling differential expression analysis via the Likelihood Ratio Test function in DESeq2 (Clevenger et al., 2017). *P*-value was calculated based on the Benjamini and Hochberg correction with a false discovery rate of 5%. We used *P*-value < 0.05 as a cutoff for defining differentially expressed genes.

### Protein Modeling and Activity

The online software Phyre2 (Kelley et al., 2015) normal mode setting was used to predict the secondary and tertiary structures of the five studied proteins. The location of the active site and the mutational sensitivity were explored using the tool PhyreInvestigator (Yates et al., 2014).

For LIN5, we studied the protein activity *in vitro*. The reference and alternate invertase coding sequences, resulting in the Asn366Asp amino acid substitution, were optimized for tomato expression and synthetic coding regions were obtained from Invitrogen (Tieman et al., 2017). The coding sequences were then cloned into p112A1 yeast expression vector. Protein expression and enzyme activity assays were performed as previously described (Fridman et al., 2004).

## Results

### Local Association Mapping Lead to Several Known Flavor Genes

**FIGURE 1:**
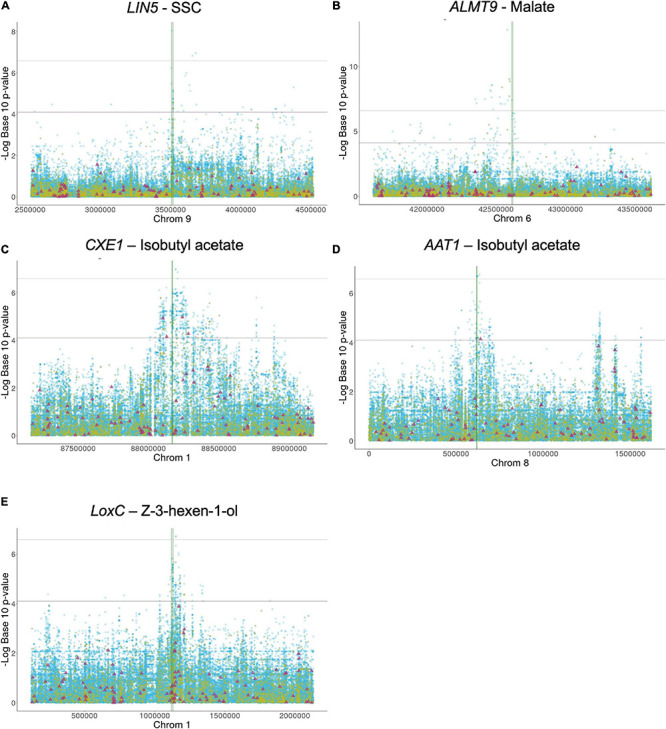
**(A-E)** Local association mapping for flavor genes and their corresponding metabolites. SNPs are plotted as blue dots, INDELs as yellow dots and SVs as purple triangles. Horizontal lines represent 0.05 and 0.01 significance thresholds. Vertical lines mark the genic region.

We compiled a list of known genes that affect tomato flavor (Table 1). For each gene, we determined whether the proposed candidate locus was significantly associated with trait variation in the Varitome collection by analyzing the coding region as well as 1 Mb upstream and 1 Mb downstream of each gene (Figure 1 and Supplementary Figure 1). The association analyses showed that variants within and near *LIN5*, *ALMT9*, *AAT1*, *CXE1*, and *LoxC* were associated with trait variation in the Varitome collection. These genes function in sugar and acid metabolism affecting taste (*LIN5* and *ALMT9*) or in volatile production affecting smell (*AAT1*, *CXE1*, and *LoxC*) (Supplementary Figure 2). The other genes listed in Table 1 did not show association with biochemical levels (Supplementary Figure 1). In addition to the metabolites displayed in Supplementary Figure 1, other metabolites from the same pathway were tested for association as well but did not show association either (data not shown).

*LIN5*- The simple sugars, glucose and fructose, are among the most important metabolites in tomato as higher levels contribute to high consumer liking (Jones and Scott, 1983; Tandon et al., 2003; Causse et al., 2010; Tieman et al., 2012). Sugars are typically evaluated by measuring the soluble solid content (SSC) which is expressed in Brix degrees. *LIN5* encodes a cell-wall invertase that hydrolyzes sucrose, and higher enzyme activity leads to increased glucose and fructose levels (Fridman et al., 2004; Zanor et al., 2009). One critical amino acid mutation between *S. pennellii* and cultivated tomato at position 348 underlies the sugar level variation between these two distantly related species. In the Varitome collection, 44 variants within or around *LIN5* were significantly associated with SSC: two SNPs in the promoter (∼2 kb upstream), one SNP in the coding region resulting in a missense mutation from asparagine to aspartate at position 366 (SL4.0ch09:3510682) and 20 variants that mapped 4 to 7 kb downstream (Figure 1A). The PVE of the most significant SNP was 14.5%. In addition, 21 significant SNPs were located further away from the gene, most of them between positions SL4.0ch09:3551616 – SL4.0ch09:4376974 (Supplementary Table 1A). Many SVs were found within and near the gene but none appeared to be associated with sugar levels (Figure 1A). The critical amino acid change between *S. pennellii* and cultivated tomato was not found in the Varitome collection.

*ALMT9*- An appropriate balance between sugars and acids is also essential for desirable tomato flavor. One major contributor to malate content is the transporter *ALMT9* that is proposed to control the accumulation of this metabolite in the vacuole (Sauvage et al., 2014; Ye et al., 2017). Higher expression of *ALMT9* leads to higher malate content in ripe fruits. Previous studies using a population of SP, SLC, and SLL implied that a 3-bp deletion in the promoter of *ALMT9* is the causative variant affecting its expression (Ye et al., 2017). In the Varitome collection, the local association mapping identified multiple highly associated variants within or around the gene (Figure 1B). A total of 66 significant variants were confined to an interval of ∼100 kb upstream of *ALMT9*. In the genic region, we found four significant SNPs, one resulting in a synonymous mutation in the second exon (SL4.0ch06:42613870) and three in the second intron (Supplementary Table 1B). The PVE of the most significant SNP was 32.7%. The 3-bp deletion in the promoter was found in 9 accessions but was not associated with malate levels in the Varitome collection.

*CXE1* and *AAT1*- Tomato flavor is highly influenced by the fruit aroma, characterized by volatile content. Acetate esters confer fruity or floral scent and are liked in high quantities in fruits such as banana, apple and melon. In tomato however, acetate esters are undesirable volatiles (Goulet et al., 2012). Acetate ester levels are controlled by a feedback loop comprised of a carboxylesterase, CXE1, and an alcohol acyltransferase, AAT1 (Goulet et al., 2012, 2015). AAT1 synthesizes acetate esters using an alcohol as precursor, whereas CXE1 catalyzes the reverse reaction (Supplementary Figure 2). The cloning of the genes revealed two different transposable elements that had integrated in the promoter of *CXE1* in SP and SLL. The transposon insertions appeared to lead to higher expression of *CXE1* in cultivated tomato compared to *S. pennellii*, thereby reducing acetate ester content (Goulet et al., 2012). For *AAT1*, on the other hand, the polymorphisms described in a previous study were several SNPs resulting in missense mutations leading to a less active protein in SLL compared to *S. pennellii* (Goulet et al., 2015). Lower AAT1 enzyme activity leads to lower levels of acetate esters in the fruit. In the Varitome collection, we selected isobutyl acetate as a proxy for all acetate esters to determine how genetic variation affected volatile levels.

At the *CXE1* locus, the local association mapping in the Varitome collection identified an interval of ∼500 kb (Figure 1C) with 650 variants that were significantly associated with isobutyl acetate levels. They included 597 SNPs, 49 INDELs and four SVs (Supplementary Table 1C). The PVE of the most significant SNP was 14.9%. Three SNPs were in the *CXE1* coding region (SL4.0ch01:88169422, SL4.0ch01:88169774 and SL4.0ch01:88169988), two resulted in missense mutations from serine to glycine at amino acid position 94 and from valine to glycine at position 211, respectively. The SVs were three deletions of 445 bp, 3.3 and 4.8 kb and one duplication of 7.0 kb (Supplementary Table 2). In nearly all cases, these four SVs were completely linked. The closest significantly associated SV was 40 kb upstream of the start site of transcription that could act as an open chromatin region affecting gene expression. Alternatively, the associated amino acid changes might alter the activity of the protein. All accessions in the Varitome collection carried the transposons in the *CXE1* promoter.

At the *AAT1* locus, an interval of 200 kb around the gene was highly associated with the phenotype in the Varitome collection (Figure 1D). The variants included 148 SNPs, three INDELs and one SV (Supplementary Table 1D). The PVE of the most significant SNP was 14.4%. Fourteen SNPs were located within the gene, including eight in the UTRs, two in introns and four resulting in missense mutations. The amino acid changes were from serine to proline at position 24, from phenylalanine to valine at position 161, and from threonine to isoleucine at positions 354 and 398. These four amino acid changes were also found between *S. pennellii* and cultivated tomato (Goulet et al., 2015). A significant 401-bp deletion was found ∼20 kb downstream the gene, which could affect gene expression. In addition, 54 SNPs were located nearly 1 Mb downstream of the gene, but their association was likely due to LD (average *R*^2^ value of 0.28, ranging from 0.12 to 0.55).

*LoxC*- Lipid-derived volatiles are also significantly associated with consumer liking as they contribute to flavor intensity (Tieman et al., 2012). Several enzymes in the biosynthetic pathway have been identified (Speirs et al., 1998; Shen et al., 2014; Li et al., 2020). LoxC catalyzes the peroxidation of linoleic and linolenic acids, producing C5 and C6 volatiles (Chen et al., 2004; Shen et al., 2014). In the Varitome collection, *LoxC* was associated with *Z*-3-hexen-1-ol, a C6 alcohol. A total of 13 INDELs and 144 SNPs were significantly associated with the volatile (Figure 1E and Supplementary Table 1E). The region that showed higher association with the phenotype was found at the 3′ end of the gene, specifically in the two last exons and the last intron. Of the 53 variants within the gene, 44 were located in introns and nine in exons. The PVE of the most significant SNP was 14.6%. Three amino acid changes were found: from valine to isoleucine at position 580, from glycine to alanine at position 598 and from threonine to leucine at position 607. In addition, a large interval of about 200 Kb downstream of the gene was associated with volatile levels, including a deletion of ∼8 Kb.

### Genetic Diversity for Flavor Genes in the Varitome Collection

#### LIN5

**FIGURE 2:**
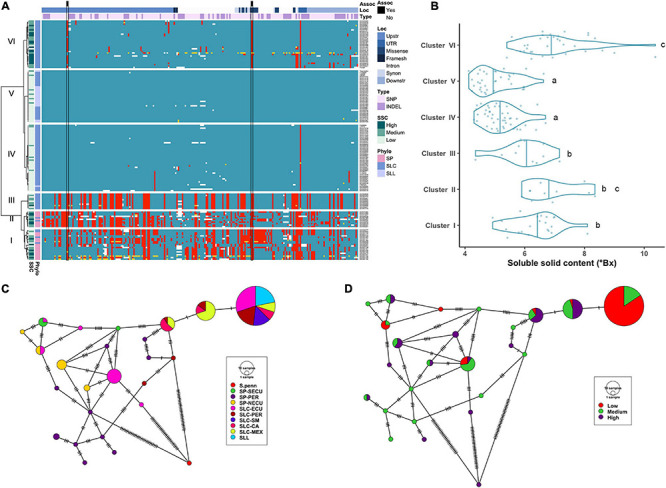
Haplotype analysis of *LIN5* locus. **(A)** Heatmap representing the genotypes of accessions (rows) for the polymorphisms identified (columns). Reference genotype are represented in blue, alternate in red, heterozygous in yellow and missing data in white. **(B)** Violin plots of the SSC content in the Varitome collection, classified by haplotype cluster. **(C)** Haplotype network classified by the phylogenetic classification of the accession. Each circle represents a haplotype, its size is proportional to the number of accessions carrying that haplotype, and lines across the edges represent mutational steps **(D)** Haplotype network classified by the SSC content.

The evolution of the *LIN5* locus may provide insights into how selection for flavor or lack thereof were part of the tomato domestication syndrome. To determine the evolution of this locus, we identified the haplotypes from the regions flanking (3 kb upstream and 1 kb downstream) and covering the *LIN5* gene. A total of 228 variants were identified at the locus (Supplementary Table 3A), of which 76 were INDELs (ranging from 1 to 97 bp), 152 were SNPs and none were SVs comprised of 100 bp or more. Most variants (60.5%) were found in the regulatory regions, defined as sequences that are upstream and downstream of the transcription start and termination site of the gene, and in the UTRs (Figure 2A). Within the gene, we identified 18 non-synonymous mutations, including 15 that resulted in amino acid changes, one in-frame deletion of five amino acids, one affecting a splicing site and one frameshift mutation leading to a presumptive null. Clustering of haplotypes into six groups revealed some association with population origins (Figure 2A). All SP were found in Clusters I and II, and both included six SLC. Cluster I mainly consisted of Ecuadorian accessions, while Cluster II consisted of Peruvian accessions. Cluster III grouped 11 SLC-ECU that shared many of the non-reference alleles found in SP. Although multiple haplotypes were observed, many of the variants were in LD with each other (Supplementary Figure 3). The remaining three clusters were similar to the Heinz 1706 reference haplotype. Cluster IV represented SLC with diverse geographical origin with three or less variants compared to the reference genome. Cluster V included SLL and a subset of SLC, primarily from Ecuador and San Martin, Peru. And lastly, Cluster VI consisted of SLC from Central America. This cluster showed the non-reference allele at three positions in nearly all accessions: a SNP at 2.7 kb upstream the transcription start site, a non-synonymous replacement in the second exon and a SNP in the 3′-UTR. The latter was also identified as a non-reference SNP in all Cluster IV accessions.

Average SSC values for each of the 6 haplotype clusters showed that Cluster IV and V displayed the lowest SSC values whereas Cluster VI and to a lesser extent Cluster II displayed the highest SSC values and Clusters I and III presented intermediate SSC values (Figure 2B). Surprisingly, only a few polymorphisms were found between Clusters IV through VI, yet Cluster VI showed the highest SSC values. Two of the significantly associated SNPs (SL4.0ch09:3505480 and SL4.0ch09:3519565) were fixed for the alternate allele in Clusters I, II, III and VI and for the reference allele at Clusters IV and V, the latter resulting in the amino acid change at position 366 (Supplementary Table 1A). An in-frame deletion resulting in a loss of five amino acids (positions 343-347) was found in 21 SP accessions belonging to Clusters I and II. This deletion could have an impact on protein activity, since an amino acid change in the adjacent position 348 was shown to be relevant in *S. pennellii* introgression line (Fridman et al., 2004). In the Varitome collection, we detected a novel frameshift mutation, which caused a loss of the start codon. This allele was found in only two accessions in Cluster VI that showed average SSC levels. Glucose and fructose levels showed the same trend as SSC, with both sugars being highest in Clusters II and VI and lowest in Clusters IV and V (data not shown).

We constructed haplotype networks using the coding sequence of *LIN5* and determined their association with the phylogenetic groups previously determined in the Varitome collection (Razifard et al., 2020; Figure 2C). Using *S. pennellii* as an outgroup, we identified 24 haplotypes demonstrating a high level of genetic diversity. The most common haplotype was identical to the reference genome, and was found in all SLL and diverse SLC populations. Only one to two mutations differentiated this haplotype from the second and third most common haplotype that were represented by SLC MEX, SLC-CA and SLC-PER. Another common haplotype was found in SLC-ECU and was closely related to the SP-NECU haplotypes. The Peruvian SP haplotypes were unique with one accession being the most ancestral haplotype. We plotted the same haplotype network to the sugar levels from high to medium to low (Figure 2D). Many ancestral SLC-MEX and SLC-CA haplotypes were associated with higher SSC values. Low SSC levels were predominant in accessions carrying the most common and reference genome haplotype, differing by only one nucleotide variant in the coding region.

#### ALMT9

**FIGURE 3:**
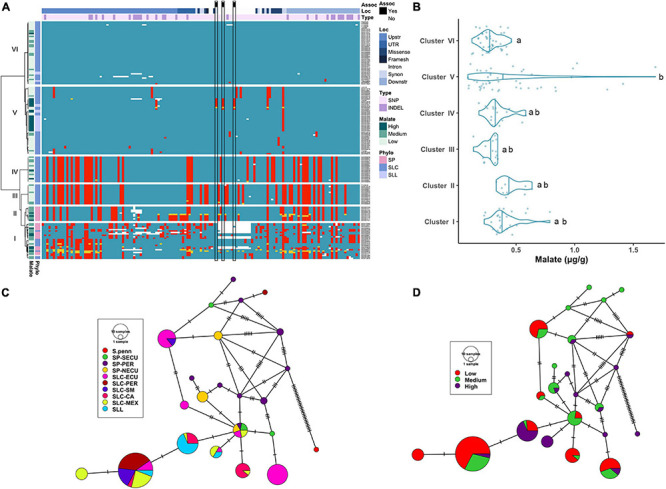
Haplotype analysis of *ALMT9* locus. **(A)** Heatmap representing the genotypes of accessions (rows) for the polymorphisms identified (columns). Reference genotype are represented in blue, alternate in red, heterozygous in yellow and missing data in white. **(B)** Violin plots of the malate content in the Varitome collection, classified by haplotype cluster. **(C)** Haplotype network classified by the phylogenetic classification of the accession. Each circle represents a haplotype, its size is proportional to the number of accessions carrying that haplotype, and lines across the edges represent mutational steps **(D)**. Haplotype network classified by the malate content.

For the *ALMT9* gene, 112 SNPs and 31 INDELs (ranging from 1 to 28 bp) were identified (Supplementary Table 3B). The variants were distributed predominantly in regulatory regions and UTRs (71.3%) and introns (14.0%). Of those that were in the coding region, 12 were non-synonymous, including a SNP that was predicted to affect splicing. The haplotype clustering analysis showed that all SP and some SLC-ECU were found in Clusters I and II (Figure 3A). Cluster I contained multiple haplotypes, indicating high genetic diversity among these accessions. A deletion of ∼2.7 kb was found in the second intron corresponding to a CopiaSL_37 retrotransposon (Ye et al., 2017) that was present in the reference genome. Most SP in Cluster I lacked the transposon insertion (Supplementary Table 2). Many SP-NECU were found in Cluster II exhibiting high genetic similarity to the SLC-ECU found in Clusters III and VI. Cluster V represented most SLL as well as SLC of diverse origin whereas Cluster VI contained SLC from diverse subpopulations.

The malate content in ripe fruits ranged from ∼0.1 to 1.7 mg/g (Figure 3B). The highest content was observed in the accessions belonging to Cluster V, although the levels were highly variable within this cluster. The median malate content was below 0.5 mg/g in all Clusters. The only two Clusters that were significantly different from one another were Cluster VI and Cluster V.

The haplotype network with the coding sequence of *ALMT9* showed 22 haplotypes (Figure 3C). The most ancestral haplotype was found in an SP-PER accession. Two common haplotypes were identified in SLC-ECU, and both differed from SP haplotypes with one unique variant. Interestingly, one haplotype appeared to have originated from SP-NECU whereas the other from SP-SECU. In the center of the network, one haplotype was shared by SP from all three geographical origins, as well as SLC-ECU and SLC-MEX. Further mutations gave rise to three additional haplotypes in SLC-CA and SLL. The most common haplotype for *ALMT9* was found in a group comprised of SLC-PER, SLC-SM, SLC-MEX and SLL. The presence of the same haplotype in multiple subpopulations indicates gene flow or lineage sorting. Seven rare SP *ALMT9* haplotypes as well as two common SLL haplotypes showed high levels of malate (Figure 3D). Most of the SLC haplotypes presented low to medium malate content, especially within the SLC-ECU.

#### *CXE1* and *AAT1*

**FIGURE 4:**
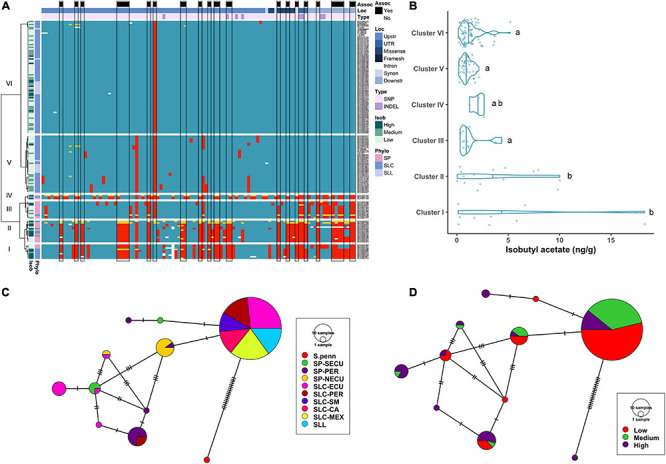
Haplotype analysis of *CXE1* locus. **(A)** Heatmap representing the genotypes of accessions (rows) for the polymorphisms identified (columns). Reference genotype are represented in blue, alternate in red, heterozygous in yellow and missing data in white. **(B)** Violin plots of the isobutyl acetate content in the Varitome collection, classified by haplotype cluster. Each circle represents a haplotype, its size is proportional to the number of accessions carrying that haplotype, and lines across the edges represent mutational steps **(C)**. Haplotype network classified by the phylogenetic classification of the accession. **(D)** Haplotype network classified by the isobutyl acetate content.

The significant association of the *CXE1* and *AAT1* loci with acetate ester content indicated that causative alleles segregated in the Varitome collection (Figure 1). *CXE1* is an intronless gene of ∼1.1 kb. Most variants were SNPs (96, 92.3%) and the remaining eight were INDELs (ranging from 1 to 14 bp) (Supplementary Table 3C). Eight missense and three synonymous mutations were found in the coding region. Of the missense mutations, five were non-conservative changes. None of the variants were predicted to lead to a significant knock down of the gene, suggesting that *CXE1* might have a critical function in adaptation. In the clustering of the gene, the upstream and downstream regions showed that the SP clustered in three groups (Figure 4A). Clusters I and II contained a mixture of SP and SLC from Ecuador and Peru respectively. Cluster III featured fewer polymorphisms with respect to the reference and included SP from all subpopulations. Cluster V contained mainly SLC-CA and seven SLL. Two variants were conserved in Cluster V, whereas 13 SNPs showed low allelic frequency in the population. Cluster VI was the largest group (78 accessions) and, compared to the reference genome, carried only one conserved SNP located ∼2 kb upstream of the gene.

Even though the normalized data showed association to isobutyl acetate levels at the *CXE1* locus, the distribution of actual levels was skewed toward 0, with ∼50% of the accessions showing less than 1 ng/g of the volatile (Figure 4B). However, a few accessions produced as high as 18 ng/g of the volatile. Accessions producing the highest content of isobutyl acetate were found in Clusters I and II, although the range within each cluster was large. Clusters III, V and VI showed low content of isobutyl acetate, with a few outliers reaching ∼5 ng/g.

The coding region haplotype network showed 10 classes. The most common haplotype (124 accessions) was found in all SLL, SLC-MEX, and SLC-SM as well as subsets from the other subpopulations (Figure 4C). Only one mutation differentiated the most common haplotype from SP-NECU and other unique SP haplotypes. Four haplotypes were associated with high isobutyl acetate content and they were represented predominantly by SP-NECU and SLC-ECU (Figure 4D). The most common haplotype included accessions that produced low (53%) as well as medium to high (47%) isobutyl acetate levels.

**FIGURE 5:**
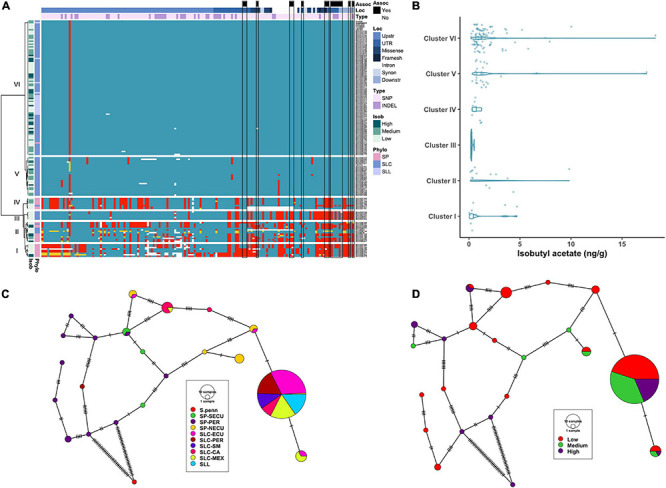
Haplotype analysis of *AAT1* locus. **(A)** Heatmap representing the genotypes of accessions (rows) for the polymorphisms identified (columns). Reference genotype are represented in blue, alternate in red, heterozygous in yellow and missing data in white. **(B)** Violin plots of the isobutyl acetate content in the Varitome collection, classified by haplotype cluster. **(C)** Haplotype network classified by the phylogenetic classification of the accession. Each circle represents a haplotype, its size is proportional to the number of accessions carrying that haplotype, and lines across the edges represent mutational steps **(D)**. Haplotype network classified by the isobutyl acetate content.

The cluster analysis of the *AAT1* locus encompassed 167 variants including 128 SNPs, 37 INDELs (ranging from 1 to 59 bp) and two SVs (Supplementary Tables 2, 3D). A relatively high proportion of these variants affected the protein sequence, resulting in missense (all SNPs) and four frameshift mutations (two SNPs, one INDEL and one SV) (Figure 5A). Four clusters each carried few accessions whereas Cluster VI was very large and identical to the reference genome except for one SNP that was located ∼2.8 kb upstream of the coding region (Figure 5A). Cluster I was genetically diverse, featuring many non-conserved polymorphisms, and was composed of SP-SECU and SP-PER. Cluster II was composed of SP from all subpopulations and a few SLC-ECU. Cluster III carried six SLC-CA where the upstream region was more similar to the reference genome than the gene and the downstream region. Cluster IV was represented by SP-NECU with high genetic similarity among the accessions. Cluster V contained SLC from Central America and Ecuador which had a similar haplotype compared to the reference, with only seven non-conserved polymorphisms. Cluster VI included all SLL and SLC from all subpopulations. Curiously, BGV006775, an SP-NECU, was found in this cluster, indicating most likely gene flow between SLC and SP accessions.

Although no significant differences in isobutyl acetate content were observed among the *AAT1* gene clusters (Figure 5B), interesting correlations between specific haplotypes and metabolite levels were noted. For example, all accessions in Cluster III carried a duplication of 13 nucleotides in the second exon that resulted in a frameshift at position 327 affecting ∼25% of the protein (Supplementary Table 3D); the average content of isobutyl acetate for accessions in Cluster III was very low, likely due to abolished activity of the enzyme (Figure 5B). Similarly, two SP_NECU from Cluster IV, which also showed low content of isobutyl acetate, carried a deletion of ∼850 kb within the gene resulting in the knock-out of the gene.

The haplotype network using the coding sequence identified 21 haplotypes, 12 of which were unique (Figure 5C). On the left side of the network, we found 10 rare haplotypes represented by SP-PER accessions and some SP-SECU. Surprisingly, a rare haplotype was found in one SLC-PER that was quite distinct from all other SLC and closer to SP-PER by six mutations. All SLL and most SLC carried the most common haplotype and differed by one mutation from a subset of SP-NECU and SLC-ECU. Isobutyl acetate levels did not show a clear pattern of distribution in the haplotype network (Figure 5D). About half of the rare haplotypes were associated with low isobutyl acetate levels. Similarly, the most common haplotype showed a mixture of high, medium and low values for isobutyl acetate.

Since AAT1 and CXE1 act in a feedback loop to control acetate ester levels, different haplotypes in one of the genes could explain the variation in clusters in the other gene. Therefore, we analyzed the haplotype distribution of each locus in the background of the most common haplotype at the other locus (Cluster VI). When selecting the accessions from Cluster VI for *AAT1*, the variation of *CXE1* explained the high content of isobutyl acetate in seven accessions from Clusters V and VI (Supplementary Figures 4A,B). These accessions shared two non-synonymous SNPs (Ser94Gly and Val211Gly), two INDELs and one SNP in the 3′-UTR and several SNPs in regulatory regions. Conversely, when the most common *CXE1* haplotype is fixed, the *AAT1* locus contributed to very low levels of isobutyl acetate, as observed in five accessions from Clusters III-VI (Supplementary Figures 4C,D).

### LoxC

**FIGURE 6:**
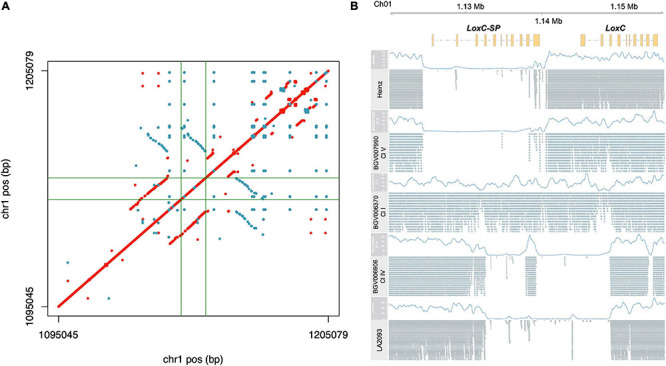
Characterization of the duplication in LoxC locus. **(A)** Dotplot resulting the pairwise comparison of *LoxC* ± 50 kb in the assembly PAS014479_MAS1.0. Each dot corresponds to an identical match of 50 bp, red in the positive strand and blue in the reverse strand. The gene coordinates are delimited by green lines. **(B)** Alignment of five representative accessions against the PAS014479_MAS1.0 assembly at *LoxC* locus, including the coverage data (blue line) and the Illumina reads.

For *LoxC*, read mapping indicated an unusual high level of apparent heterozygosity in SP accessions and we sought to explore that first (Supplementary Figure 5A and Supplementary Table 3E). Because such extensive heterozygosity is rare in tomato, we hypothesized that this signal actually indicated a duplication with respect to the reference genome. In this scenario, duplication heterogeneity appears as heterozygosity when paralogous reads are mismapped to the single-copy reference locus. Using the previously established long-read assembly of PAS014479 accession, an SP-PER (Alonge et al., 2020), we identified a duplication of ∼15 kb, covering the entire *LoxC* gene (Figure 6A). A third partial copy in the reverse strand, which appeared to have arisen from an inversion, was found downstream *LoxC*. This sequence was also found in the Heinz reference genome (data not shown) and did not appear to encode another paralog of *LoxC* since no gene model was predicted. To check whether this duplication was correlated with heterozygosity signal, we analyzed the alignments of a subset of representative accessions using PAS014479 as the reference. The reference genome and accessions with a similar haplotype at this locus, e.g., BGV007990, carried a deletion of ∼15 kb immediately upstream *LoxC* in accordance with the duplication coordinates, while the apparent heterozygous accessions, e.g., BGV006370, lacked the deletion (Figure 6B). In addition, alternative structural variants were found in certain SLC-ECU accessions, e.g., BGV006906, and this was shared with another sequenced accession, LA2093 (Wang et al., 2020). Altogether, we propose that *LoxC* experienced an ancestral tandem duplication in SP, which later diverged generating two copies of the gene with 91% protein identity. The non-reference copy of *LoxC*, *LoxC-SP*, was deleted in most SLC and SLL, and another deletion partially affecting both *LoxC* and *LoxC-SP* appeared in a small group of SLC-ECU.

*LoxC-SP* was found in 28 accessions (Supplementary Table 4), including SP from both Peru and Ecuador and several SLC-ECU. The average *Z*-3-hexen-1-ol content in accessions containing both *LoxC* and *LoxC-SP* was 16.4 ng/g, whereas the accession carrying exclusively *LoxC* showed 25.6 ng/g of the volatile (Supplementary Figure 5B). Although this difference is significant (*p*-Value = 0.021), *Z*-3-hexen-1-ol content varied within each group, with a range from 0.01 to 70.61 and 0.14-98.77 ng/g when the duplication was present and absent, respectively. Therefore, additional genetic variation at the locus was likely responsible for the phenotypic variation found within the groups. We performed the association mapping at the locus using the subset of accessions containing exclusively *LoxC* and obtained seven significant SNPs (Supplementary Figure 5C and Supplementary Table 1F). All significant SNPs were still significant when analyzing the entire Varitome collection. Three of the significant SNPs were located upstream the gene, one in the first intron and other three downstream the gene.

When excluding the accessions carrying *LoxC-SP*, we identified 426 variants, of which 332 were SNPs, 92 were INDELs and 2 were SVs (Supplementary Table 3F). Among them, two mutations were predicted to affect splicing, and 15 SNPs were missense mutations. The SVs were two deletions of 291 bp and 795 bp in the first intron, present in two and three accessions respectively.

**FIGURE 7:**
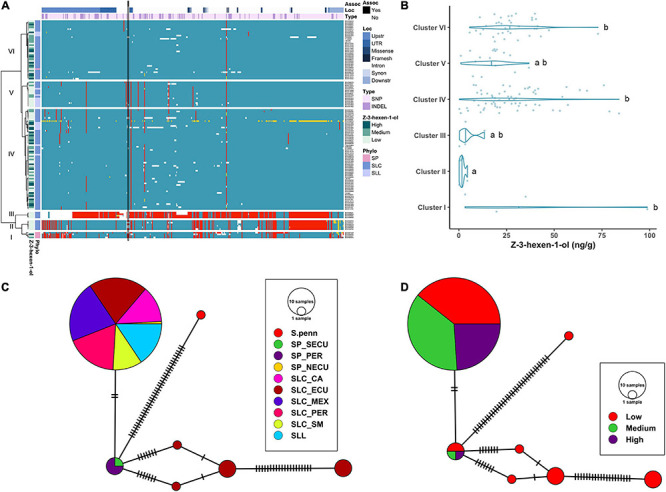
Haplotype analysis of *LoxC* locus for accessions without duplication. **(A)** Heatmap representing the genotypes of accessions (rows) for the polymorphisms identified (columns). Reference genotype are represented in blue, alternate in red, heterozygous in yellow and missing data in white. **(B)** Violin plots of the Z-3-hexen-1-ol content in the Varitome collection, classified by haplotype cluster. **(C)** Haplotype network classified by the phylogenetic classification of the accession. Each circle represents a haplotype, its size is proportional to the number of accessions carrying that haplotype, and lines across the edges represent mutational steps **(D)**. Haplotype network classified by the Z-3-hexen-1-ol content.

The haplotype analysis produced three clusters containing few, divergent accessions and three large clusters similar to the reference (Figure 7A). Cluster I was composed of SP accessions, and Clusters II and III of SLC-ECU. Of these three clusters, Cluster III was the most divergent with respect to the reference genome. Clusters I and II shared most of the variants, except those located at the 3′ end of the gene. Cluster III presented a putative deletion in the promoter, ∼500 bp upstream of the start site, which may impact *LoxC* expression. Clusters II and III featured low *Z*-3-hexen-1-ol content, suggesting that the polymorphisms at the 3′ end of the gene could have an impact on the phenotype (Figure 7B). Cluster IV was the largest group, containing 7 SLL and 56 SLC from all subpopulations, whereas most SLL were grouped in Cluster V. Both clusters showed several polymorphisms compared to the reference genome, although none of them impacted protein sequence. Lastly, Cluster VI was the most similar to the reference genome and was comprised of SLC from all subpopulations. Clusters IV and VI presented on average higher volatile content than Cluster V.

The haplotype network using the coding sequence generated one common haplotype shared by SLL and many diverse SLC (Figure 7C). Only two polymorphisms differentiated this haplotype from the SP-PER haplotype, identified as the most ancestral haplotype. Another four divergent haplotypes were found exclusively in SLC-ECU. The latter were carried exclusively by accessions with low *Z*-3-hexen-1-ol content, indicating that those mutations could have a role in protein activity (Figure 7D). In contrast, the most common haplotype contained similar proportions of low, medium and high volatile producers, suggesting that the difference between these accessions was likely regulatory in nature.

### Distribution of Genetic Variation in Flavor Genes

To estimate the genetic diversity of these five flavor-related genes among subpopulations, we estimated the nucleotide diversity (Supplementary Figure 6). When considering overall genetic diversity, SP-PER is the most diverse group, followed by other SP and SLC-ECU, which showed similar values. In general, genetic diversity was reduced in other SLC subpopulations, and further reduced in SLL, in agreement with whole-genome genetic diversity (Razifard et al., 2020). However, specific subpopulations showed higher levels of diversity in some genes, e.g., SLC-SM for *ALMT9* and SLC-CA for *AAT1*, likely due to gene flow between these groups and SP.

For all five genes, non-coding regions carried the highest proportion of genetic diversity, as expected (Supplementary Figure 6). Synonymous and non-synonymous π estimates were similar overall, yet in some cases non-synonymous genetic diversity was predominant (e.g., *ALMT9* in SLC-MEX, *AAT1* in SP-SECU and SP-PER and *CXE1* in SLC-ECU, SLC-PER and SLC-CA), which may suggest positive selection on non-synonymous mutations with beneficial impact.

We hypothesized that some potentially valuable haplotypes may have been left behind during domestication and improvement of tomato. To test whether novel haplotypes conferring superior flavor found in the Varitome collection were absent in cultivated tomato, we selected a representative subset of cultivated accessions for which sufficiently high-quality sequencing data were publicly available. As expected, for all genes except *LoxC*, the number of polymorphisms found in cultivated tomato was lower than in the Varitome collection (Supplementary Table 5). Furthermore, most of the accessions carried none or few alternate alleles (<5 variants). Around one to four accessions showed a divergent haplotype with most variants homozygous for alternate allele, probably resulting from introgressions of genomic regions from related wild species. The most common haplotype of the known flavor genes did not appear to be the optimal haplotype. For *LIN5*, the best haplotype (Cluster VI) was not found in cultivated tomato. Five accessions carried the alternate allele of the two associated variants from this cluster, but in combination with other polymorphisms. For *ALMT9*, the desirable haplotype associated with lowest malate content (Cluster VI) was present in both the Varitome collection and cultivated tomato. For *CXE1*, the best haplotype was difficult to discern. One of the likely beneficial haplotypes in *CXE1* (Cluster VI) was found in cultivated tomato. For *AAT1*, the best haplotypes (Clusters III and VI) were absent from cultivated tomato; only one accession from Tunisia carried a likely beneficial haplotype. For *LoxC*, three haplotypes were associated with higher levels of *Z*-3-hexen-1-ol (Clusters I, IV, and VI) and only Cluster VI haplotype was present in cultivated tomato.

Haplotype analyses showed that SLL had no unique haplotypes. Hence, the haplotypes of flavor genes that characterize cultivated tomato appeared to have come from standing genetic variation present in ancestral populations. Novel mutations in flavor genes rarely appeared during domestication according to the results at these five genes. Since only certain haplotypes were selected and those were now nearly fixed in cultivated tomato, SLC accessions from South and Central America continues to be a good source of improved haplotypes at these loci.

### Gene Expression of Flavor Genes

**FIGURE 8:**
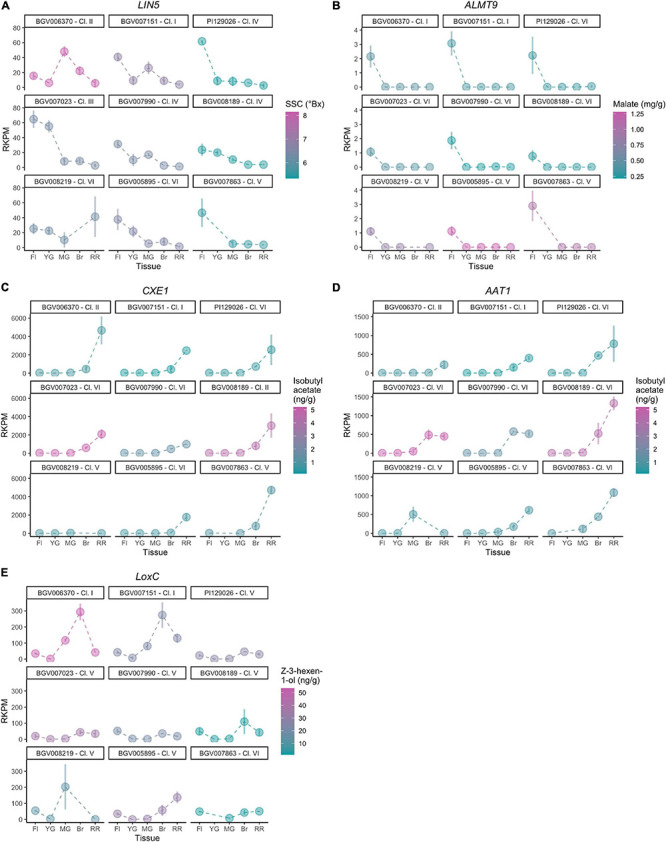
**(A–E)** Gene expression of nine representative accessions for flavor-related genes.

For each known gene in a metabolic pathway, its protein activity (Fridman et al., 2004; Goulet et al., 2015) and gene expression (Goulet et al., 2015) collectively contribute to the accumulation of the metabolite. To evaluate whether expression of the studied genes was associated with the accumulation of metabolites, we performed a transcriptome analysis of nine diverse accessions from different phylogenetic groups presenting a range of metabolite content (Table 2). Five developmental stages of fruit development were selected, from flower at anthesis to ripe red fruit, for insights into gene expression dynamics. Since there are two variables (genotype and developmental stage), we used linear modeling instead of pairwise comparison to identify differentially expressed genes. In brief, the Likelihood Ratio Test is used to provide a *P*-value for each gene for identifying differential expression based on a cut-off value of 0.05 (Clevenger et al., 2017). Although the five studied genes were all involved in fruit flavor, the expression patterns observed were different among the accessions that were used in the study (Figure 8). The raw mapping data were listed in Supplementary Table 6.

**TABLE 2:** Accessions used for transcriptomic analysis and corresponding metabolite levels.

**Accession**	**Subpopulation**	**SSC (°Bx)**	**Malate (μg/g)**	**Isobutyl acetate (ng/g)**	**Z-3-hexen-1-ol (ng/g)**
BGV006370	SP_PER	8.15	0.45	0.73	53.44
BGV007151	SP_SECU	6.90	0.35	0.13	23.59
PI129026	SLC_ECU	5.33	0.29	0.36	26.01
BGV007023	SLC_ECU	6.40	0.42	5.21	37.07
BGV007990	SLC_PER	6.43	0.21	1.36	20.11
BGV008189	SLC_PER	5.37	0.25	4.52	1.02
BGV008219	SLC_MEX	6.25	0.84	0.71	11.60
BGV005895	SLC_MEX	6.60	1.28	0.75	32.00
BGV007863	SLL	5.47	1.02	0.92	1.04

For *LIN5*, the expression dynamics varied substantially between accessions (Figure 8A) which was confirmed by the calculated *P*-value of 7.21 - 10^–13^. The flower stage showed the highest expression level in most accessions. BGV006370, an SP-PER accession in haplotype Cluster II, featured high SSC and showed the highest expression of *LIN5* in mature green fruit. The same pattern was observed but to a lesser extent in BGV007151, an SP-SECU accession. In accessions that accumulated lower SSC, *LIN5* expression peaked at the flower stage. BGV008219 showed a different expression pattern that peaked at the ripening stage, albeit that the replicates were variable. These data suggested that the timing of expression may be relevant for fruit sugar content which could have changed during domestication.

For *ALMT9*, the expression pattern was similar in all accessions (Figure 8B) with a calculated *P*-value of 1, with low expression that peaked at the flower stage. Of the nine accessions in the expression analysis, only one (BGV008219) carried the 3-bp INDEL in the promoter described before as likely causative (Ye et al., 2017). However, BGV008219 *ALMT9* expression levels did not differ dramatically from any of the other accessions. Moreover, malate content did not correlate to expression levels among these nine accessions. For example, of the four accessions in Cluster VI, two accessions showed higher expression, but the malate content was still low. The lack of correlation between gene expression and malate content could be due to the limited number of samples analyzed and/or genetic background effects. The expression of *ALMT9* could also be restricted to a very specific tissue or stage of development, which would impede to reach conclusions from the current experiment. In addition, any of the missense mutations could alter protein activity and cause the observed phenotype.

For *AAT1* and *CXE1*, we observed a similar pattern of expression in most accessions, showing low expression in flower and the first stages of fruit development. Expression started to increase at breaker and peaking in ripe fruits (Figures 8C,D). However, the levels of expression in red ripe fruit varied greatly among accessions, therefore both of *AAT1* (*P*-value of 3.01 - 10^–10^) and *CXE1* (*P*-value of 2.77 - 10^–12^) were categorized as differentially expressed genes in linear modeling analysis. In most cases, the expression of *AAT1* and *CXE1* was equally high; for example, BGV008189 showed the highest expression for *AAT1* and also one of the highest for *CXE1*. However, in the SP accessions BGV007151 and BGV006370, expression of *AAT1* was low, limiting the synthesis of isobutyl acetate, whereas expression of *CXE1* was high, further enhancing the degradation of the limited amount of the volatile. The two accessions that showed high *CXE1* expression in ripe fruit showed medium to low isobutyl acetate content, which fits the hypothesis of these esters to be catalyzed at a high rate. Four SLC contained in Cluster VI showed lower *CXE1* expression on average, yet the metabolite content was variable within the group. *AAT1* expression was lower (<500 RKPM) in the two SP accessions, from Clusters I and II, than in accessions from Cluster VI, the most common haplotype (∼1000 RKPM).

The expression levels of *LoxC* were variable across accessions as indicated by a *P*-value of 1.22 - 10^–18^, although the dynamics were similar. In most of them, the expression was low at flower and young fruit, increased gradually until it peaked at breaker and then slightly reduced in ripe red fruits (Figure 8E). *LoxC* expression at breaker stage was nearly tripled in the two SP accessions carrying the duplication, suggesting a gene dosage effect. No general relationship among gene expression and *Z*-3-hexen-1-ol content was observed. However, BGV006370 presented the highest expression level at breaker as well as the highest *Z*-3-hexen-1-ol content and the SLL accession BGV007863 showed low levels of both expression and metabolite level.

### Effects on Protein Structure

Several variants that alter protein sequences were identified in the five known flavor genes. To estimate how these variants could alter the protein structure and function, we predicted the 3D model for each protein and the effect of missense mutations.

The best model template for LIN5 was a cell-wall invertase from *Arabidopsis thaliana* (Supplementary Figure 7 and Supplementary Table 7). The prediction was of high quality, and the identified domains were members of the glycosyl hydrolases family 32. One transmembrane domain was predicted between positions 524-539. Of the 15 missense mutations, only one was predicted to have a high impact on protein structure, a change from Phenylalanine to Leucine in position 318 in the active site (Table 3). The in-frame deletion of five amino acids from 343 to 347 positions affected two amino acids predicted to be part of the active site; however, their mutational sensitivity was considered low. Therefore, it was unclear whether this INDEL could have a measurable impact on protein structure and activity. The change from Asparagine to Aspartate at position 366 was the most highly associated SNP in our analyses as well as former studies (Fridman et al., 2004; Tieman et al., 2017), yet it was predicted to have minimum effect on protein structure. These two variants of the LIN5 protein when overexpressed in tomato revealed that plants overexpressing the alternate version of the protein had higher sugar levels than those expressing the reference version of the protein (Tieman et al., 2017). To determine the biochemical basis for this phenotype, we expressed the two variants of the LIN5 protein in yeast. The alternate version of the protein containing Asp at position 366 exhibited higher activity with respect to sucrose substrate than the reference version of LIN5 (Supplementary Table 8).

**TABLE 3:** Amino acid changes and predicted impact in protein structure.

**Protein**	**Mutation**	**Impact severity in the protein structure**	**Pocket**	**Associated with phenotype**
LIN5	Phe21Tyr	1		
	Ile208Val	1		
	Tyr265His	2		
	Met290Val	1		
	Phe318Leu	7	*	
	Asn366Asp	1		*
	Leu373Val	1		
	Lys385Arg	1		
	Leu390Trp	2		
	Lys393Asn	1		
	Leu422Phe	2		
	Val440Leu	1		
	Val458Leu	1		
	Ser494Thr	1		
	Asn498Asp	1		
ALTM9	Lys47Asn	2		
	Val86Ile	1		
	Val152Phe	3		
	Gly215Ser	1		
	Pro277Leu	3		
	His307Arg	1		
	Tyr406Asn	3		
	Glu412Ala	2		
	Leu458Ser	2		
	Arg504His	2		
	Ala554Val	2		
CXE1	Gln66Leu	2		
	Gly77Ser	5		
	Ser94Gly	5		*
	Phe154Ile	5		
	Gly200Asp	2	*	
	Val211Gly	2		*
	Leu214His	2		
	Ser266Tyr	3		
AAT1	Ile4Thr	2		
	Ser24Pro	1		*
	Leu41Phe	1	*	
	Leu60Pro	2		
	Lys88Arg	1	*	
	Tyr123Cys	2		
	His129Arg	3		
	Ile145Val	1		
	Phe161Val	5		*
	Asn176Lys	2		
	Cys209Phe	2		
	Val245Phe	1		
	Arg270Cys	6		
	Leu284Phe	3		
	Thr354Ile	1		*
	Thr398Ile	1		*
LoxC	Leu43Ile	2		
	Ile52Thr	1		
	Glu57Gln	1		
	Val72Leu	1		
	Pro178Ser	1		
	Leu190Ile	2		
	Ser191Pro	1		
	Asn264Lys	1	*	
	Gln294Lys	1		
	His337Gln	2		
	Asn366Asp	1		
	Val580Ile	1		*
	Gly598Ala	2		*
	Thr607Leu	3		*

*Indicates the amino acid changes affecting the pocket of the enzyme (column 4) and/or significantly associated with the phenotype (column 5).

For ALMT9, the model presented low quality, reaching only 56.1% of confidence, on the contrary to the other models (Supplementary Figure 7 and Supplementary Table 6). The model contained seven transmembrane domains, which would be consistent with the subcellular localization of the protein in the tonoplast (Ye et al., 2017). None of the 11 missense mutations was predicted to cause a meaningful effect on protein structure (Table 3).

The best model template for CXE1 was an alpha-beta hydrolase from *Catharanthus roseus*, which covered 98% of the protein sequence (Supplementary Figure 7 and Supplementary Table 6). Three out of the eight missense mutations were predicted to produce a moderate effect on protein structure (Table 3). In addition, one of these amino acid changes, from Serine to Glycine in position 94, was significantly associated with isobutyl acetate levels, suggesting that it might alter the activity of the enzyme.

For AAT1, the best model template was a hydroxycinnamoyl-coA transferase from *Coffea canephora*, which carried a domain from a transferase family as well as one transmembrane domain between positions 257-272 (Supplementary Figure 6 and Supplementary Table 5). Two amino acid changes were predicted to cause a moderate effect on protein structure, from Phenylalanine to Valine at position 161 and Arginine to Cysteine at position 270 (Table 3). The position 161 amino acid change-causing SNP was significantly associated with isobutyl acetate levels in the local association mapping result (Figures 1, 4) and was one of the amino acid changes identified between *S. pennellii* and cultivated tomato (Goulet et al., 2015).

The best model template for LoxC was a lipoxygenase from plants. The model contained the two known domains, PLAT and lipoxygenase, that are found in these enzymes (Supplementary Figure 6 and Supplementary Table 5). Most amino acid changes were predicted to have a low impact on protein structure. However, the change from Threonine to Leucine at position 607 showed the highest likelihood of changing protein structure and the underlying SNP was highly associated with *Z*-3-hexen-1-ol (Table 3).

## Discussion

Fruit flavor is a complex trait that is genetically controlled by several independently regulated pathways (Tieman et al., 2012, 2017). For good tomato flavor, the balance of sugars and acids is complemented by the production of a specific bouquet of volatile organic compounds. Flavor is also affected by the environment and levels of certain metabolites can range from ∼20 to 80% (Bauchet et al., 2017). Some metabolic traits also show a significant interaction between genetic and environmental effects (Diouf et al., 2018). Despite environmental effects, five previously cloned genes representing four flavor pathways, were significantly associated with trait variation in the Varitome collection. This suggested that these five genes were major contributors to flavor change during the evolution of the vegetable. The domestication of tomato started with the origin of semi-domesticated SLC in South America, the northward spread of SLC and the further domestication into SLL in Mexico. Of the candidate genes examined, only *LIN5* showed evidence of having been subjected to positive selection during the final steps of domestication (Razifard et al., 2020). *AAT1* was also associated with sweeps in the transition from SP to SLC-ECU and in the sweep in the northward migrations of SLC. The lack of evidence on the positive selection on three of the five flavor genes during domestication is consistent with our view and that of others (Blanca et al., 2012, 2015; Sauvage et al., 2017), that some potentially valuable haplotypes may have been left behind during the evolution from a fully wild to a cultivated type. The selected haplotypes for *LIN5* and *AAT1* seemed to have contributed negatively to flavor, meaning they could have hitchhiked due to linkage drag with another trait in the region. Alternatively, the flavor deterioration could have been a tradeoff for improved agricultural performance, e.g., sugar content and fruit size are often inversely correlated (Georgelis et al., 2004; Prudent et al., 2009). In this case, positive selection for larger fruits would lead to fixation of haplotypes conferring lower SSC.

To determine whether the diversity in the Varitome collection is useful toward improving modern tomato flavor, we sought to find the optimal allele for each gene. For *LIN5*, an enzymatic assay from a previous study showed that the change at position 348 from Aspartate in *S. pennellii* to Glutamate in *S. lycopersicum* played a role in protein activity (Fridman et al., 2004). In the red-fruited Varitome collection, a different change from Asparagine to Aspartate at position 366, was significantly associated with sugar content (Figure 1A), consistent with findings from other GWAS (Tieman et al., 2017; Razifard et al., 2020). Protein expression studies showed that this amino acid replacement altered protein activity (Supplementary Table 8) and overexpression of the Asp^366^ *LIN5* allele in tomato increased sugar content (Tieman et al., 2017). The less desirable Asn^366^ allele is present at high frequency in SLL, and in 94.6% of the selected heirloom and modern varieties (Supplementary Table 5). Thus, the optimal allele of *LIN5* appeared to be rare in modern tomato.

For *ALMT9*, a 3-bp INDEL in the promoter was proposed to be causative to trait variation (Ye et al., 2017). This small INDEL would impact a W-box binding motif thereby affecting gene expression. In the Varitome collection, the most significant variants were three SNPs located in the second exon (synonymous) and the second intron (Figure 3). The 3-bp INDEL was not associated with the trait, possibly due to low allele frequency in the Varitome collection, which could reduce the statistical power to detect significant associations. In the subset of heirloom and modern tomatoes, this INDEL and the three SNPs were in complete LD, suggesting that the effect on the phenotype was by a combination of these variants. This haplotype found in some SLL and SLC, is thought to contribute to increased malate content in fruits, which is associated with negative flavor. Therefore, this haplotype may not be desirable in breeding programs aimed at improving flavor. In addition to its role in fruit flavor, ALMT9 contributes to Al tolerance in roots (Ye et al., 2017). None of the haplotypes found in the Varitome collection and the heirloom and modern accessions were predicted to be a gene knock-out, suggesting that a functional *ALMT9* may be essential. These findings suggest that it may be relevant for plant performance and adaptation to novel environments. However, the effect of the less tasty *ALMT9* allele on plant performance in this collection is unknown. In the Varitome collection, two novel haplotypes (Clusters III and IV) were also associated with low malate content and could be used in breeding programs for improved flavor.

The transposable elements in the promoter of *CXE1* are proposed to increase expression in red fruited tomato compared to the green fruited *S. pennellii* (Goulet et al., 2012). These transposable elements were fixed in the Varitome collection, yet differences in gene expression were still observed. For example, two accessions from Cluster II showed a 2-fold increase in expression of *CXE1* compared to accessions in Cluster VI at the ripe fruit stage (Figure 8). Several SNPs and INDELs in regulatory regions differed between these two groups, which could lead to differences in gene expression. In addition, eight missense SNPs were identified in the Varitome collection, of which only one was found in the heirloom and modern accessions (Supplementary Table 5). Haplotypes found in Clusters I and II were associated with higher acetate esters content. Since acetate esters are negatively correlated with consumer liking (Tieman et al., 2012), the Cluster I and II haplotypes were undesirable. The most common and most desirable haplotype in SLL were found in Clusters V and VI and were identical or nearly identical to the reference genome (Figure 4). In addition, a novel SP haplotype from Cluster III contributes to low acetate content and may also be used in breeding programs to enhance fruit flavor.

The *S. pennellii* AAT1 enzyme is proposed to be more active than cultivated AAT1 (Goulet et al., 2015). The specific polymorphism(s) causing the variation in acetate ester levels is not known, however. Several polymorphic SNPs leading to amino acid changes between *S. pennellii* and cultivated tomato were also segregating in the Varitome collection, three of which were significantly associated with acetate ester levels (Table 3). Interestingly, some of the polymorphisms found in *S. pennellii* were shared by SP. However, SP showed low acetate ester levels whereas *S. pennellii* showed high levels implying that these polymorphisms are inconsequential. In addition, two haplotypes that were predicted to result in a knock-out or knock-down of the gene were found. One haplotype carried a deletion of ∼850 bp affecting the coding sequence and another carried a 13-bp duplication resulting in a coding region frame shift. Both haplotypes were associated with low content of acetate esters, which is positively correlated to consumer liking. The latter polymorphisms were largely absent in the heirloom and modern varieties. Therefore, these *AAT1* knock-down haplotypes leading to reduced production of acetate esters could be easily introduced into breeding programs to contribute to flavor improvement.

The availability of improved long-read genome assemblies allowed us to resolve several SVs affecting the *LoxC* locus. A heterozygous promoter allele is reported to be associated with higher gene expression in a previous study (Gao et al., 2019). However, we found a gene duplication causing a misleading level of heterozygosity. The duplication was mainly found in SP and, on average, contributed to lower levels of *Z*-3-hexen-1-ol. The expression of *LoxC* in SP was higher, as previously reported, but this did not appear to result in higher *Z*-3-hexen-1-ol accumulation. The encoded LoxC and LoxC-SP showed only a 91% amino acid identity (data not shown), implying that these paralogs arose millions of years ago. In addition, a QTL mapping study using a RIL population derived from a cross with NC EBR-1 (only reference *LoxC* copy) and LA2093 (incomplete *LoxC* and *LoxC-SP* copies) found increases in multiple lipid-derived volatiles and apocarotenoids controlled by the NC EBR-1 haplotype (Gao et al., 2019; Wang et al., 2020). According to our findings, LA2093 suffered a deletion of ∼16 kb which fused the first three exons of *LoxC-SP* to the last eight exons of *LoxC*, with the third exon being duplicated (Figure 6B). Since the LA2093 haplotype was associated with low content of volatiles, it was conceivable that the encoding enzyme was not functional. When excluding the accessions carrying both copies of *LoxC*, the Cluster III haplotype (Figure 7) differed in most variants, suggesting that these accessions could only carry the *LoxC-SP* paralog and/or the deletion found in LA2093. Among the other reference *LoxC* haplotypes, we could not find a likely causative variant. The reference haplotype (Cluster VI) seems to be adequate for high lipid-derived volatile content (Figure 7). In addition, the haplotype found in Cluster IV might also be beneficial for flavor improvement.

Regulatory mutations are often causative of trait variation (Muños et al., 2011; Goulet et al., 2012; Ye et al., 2017). Surprisingly, we did not find a clear correlation between gene expression and metabolite levels for none of the five studied genes. This lack of correlation may be due to several biological and technical factors. On one hand, the causative variants may affect the coding region and/or UTRs instead of being regulatory, as shown for *LIN5*. On the other hand, we limited the transcriptomic analyses to nine representative accessions, which we thought to be representative of the Varitome collection, yet may not reflect completely all haplotypes. For example for *ALMT9*, only clusters I, V and VI were represented in the expression analysis (Figure 8).

To envision the use of the findings from these studies in tomato breeding programs, the beneficial haplotypes at these five loci could be introgressed through conventional breeding into cultivated germplasm and evaluated for their performance. Moreover, we showed that SLC maintained levels of genetic diversity comparable to SP at the five flavor loci even though SP is evolutionary quite distinct from SLC and instead SLC is much closer to SLL (Supplementary Figure 6). Therefore, an added benefit of using SLC accessions as donors for beneficial alleles is the reduced linkage drag of deleterious alleles that often accompanies the introgression of targeted loci from more distant wild relatives. The detailed analyses of the fruit metabolite loci permitted us to propose the likely relevant variant(s), which can be used to identify the best donor accession as well as the development of molecular markers to monitor the introgression. Once incorporated into modern accessions, the effect of these haplotypes could be directly tested and validated.

The genetic variation for each locus in the Varitome collection was large. Moreover, even within genetic clusters, we observed wide phenotypic variation, suggesting that additional genetic factors are segregating in the population for these pathways. These other genes could be previously cloned genes (albeit that they did not show association in the Varitome collection) or representing novel genes. Our collection would be an excellent material to discover new flavor genes through genetic mapping approaches.

## Data Availability Statement

The datasets analyzed for this study can be found in NCBI, accession numbers SRA: SRP150040, SRA: SRP045767, and SRA: SRP094624.

## Author Contributions

LP and EvdK conceived the study. LP, MS, MA, NT, YiZ, YoZ, and HR performed the experiments and data analyses. DT generated the metabolic data. YW generated the RNA-seq data. AF, AC, ZF, and MCS provided advice and resources. LP and EvdK drafted the original manuscript. All authors reviewed and agreed to the published version of the manuscript.

## Conflict of Interest

The authors declare that the research was conducted in the absence of any commercial or financial relationships that could be construed as a potential conflict of interest. The reviewer GD declared a past co-authorship with one of the authors AF and the reviewer CS declared a past co-authorship with several of the authors AF and DT to the handling editor.

## Footnotes

1 https://www.ncbi.nlm.nih.gov/

2 https://www.bioinformatics.babraham.ac.uk/projects/fastqc/

3 https://solgenomics.net/organism/Solanum_lycopersicum/genome

4 https://solgenomics.net

5 https://solgenomics.net/projects/tomato13

## Supplementary Material

Supplementary Figure 1 Local association mapping for flavor-related genes and their corresponding metabolites. SNPs are plotted as blue dots, INDELs as yellow dots and SVs as purple triangles. Horizontal lines represent 0.05 and 0.01 significance thresholds.

Supplementary Figure 2 Function of the known genes used in this study, as detailed previously in the literature.

Supplementary Figure 3 Linkage disequilibrium of SNPs in the gene regions.

Supplementary Figure 4 Haplotype analysis of AAT1 and CXE1 A. Heatmap of AAT1 including only accessions which belong to Cluster VI in CXE1 clustering B. Violin plots of the isobutyl acetate content classified by haplotype cluster. C. Heatmap of CXE1 including only accessions which belong to Cluster VI in AAT1 clustering D. Violin plots of the isobutyl acetate content classified by haplotype cluster.

Supplementary Figure 5 Haplotype analysis of *LoxC* locus for the complete set of accessions. **(A)** Heatmap representing the genotypes of accessions (rows) for the polymorphisms identified (columns). Reference genotype are represented in blue, alternate in red, heterozygous in yellow and missing data in white. **(B)** Violin plots of the Z-3-hexen-1-ol content for accessions carrying the duplication (*LoxC-SP* present) and without the duplication (*LoxC-SP* absent).

Supplementary Figure 6 Nucleotide diversity in the gene regions, including flanking sequences 3 kb upstream and 1 kb downstream, within each subpopulation.

Supplementary Figure 7 Protein modeling predictions of the five proteins using amino acid sequences. The predicted pocket of the enzyme is displayed in red.

Supplementary Table 1 Association mapping results. The variant ID includes a first code letter: S for SNP, I for indel and V for SV. The significant *p*-Values are highlighted in pink color.

Supplementary Table 2 Genotyping of SVs detected using Lumpy **(A)**. For all five genes and the complete Varitome collection. **(B)** For LoxC when excluding the accessions carrying LoxC-SP.

Supplementary Table 3 Genotyping table. Each column corresponds with a variant and the coordinate, reference and alternate alleles and variant annotation from SnpEff are included. Each row corresponds to an accession and the ID of the accession, Cluster at which belongs according to the haplotype clustering and subpopulation according to Razifard et al. (2020) are included.

Supplementary Table 4 Genotyping of the duplication at the *LoxC* locus using three different criteria: normalized coverage and heterozygosity when aligning against Heinz SL4.0 reference genome and detection of a deletion when aligning to the PAS014479_MAS1.0 assembly. 0 means only *LoxC* copy, 1 means both *LoxC-SP* and *LoxC* copies.

Supplementary Table 5 Genotyping results of the selected cultivated varieties at the five loci. Information about the origin and whether the variety is modern or heirloom was extracted from Tieman et al. (2017).

Supplementary Table 6 RPKM values for each biological replicate used in the expression analysis.

Supplementary Table 7 Quality parameters of the protein modeling predictions.

Supplementary Table 8 Enzymatic activity of reference and alternate LIN5 alleles.
