# Supplementary material for: Natural Genetic Diversity in Tomato Flavor Genes
Source: Front Plant Sci. 2021 Jun 4;12:642828. doi: 10.3389/fpls.2021.642828 (PMC8212054; doi:10.3389/fpls.2021.642828)

Carotenoids

PSY1 - lycopene

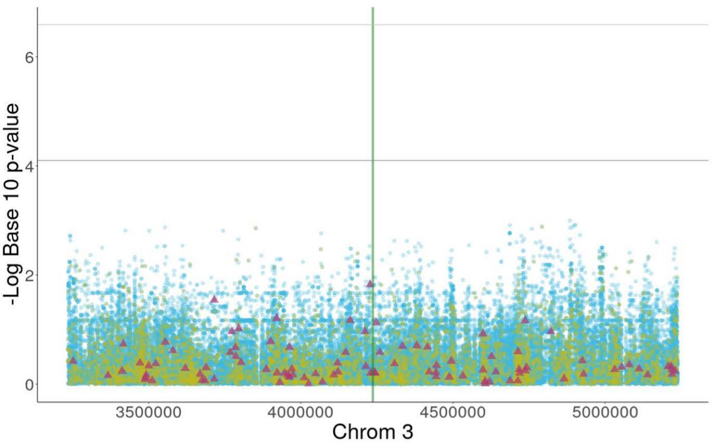

CrtISO - β-carotene

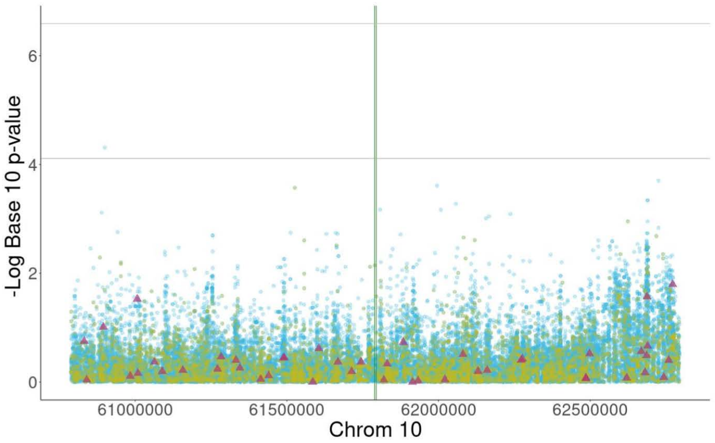

CYCB – β-carotene

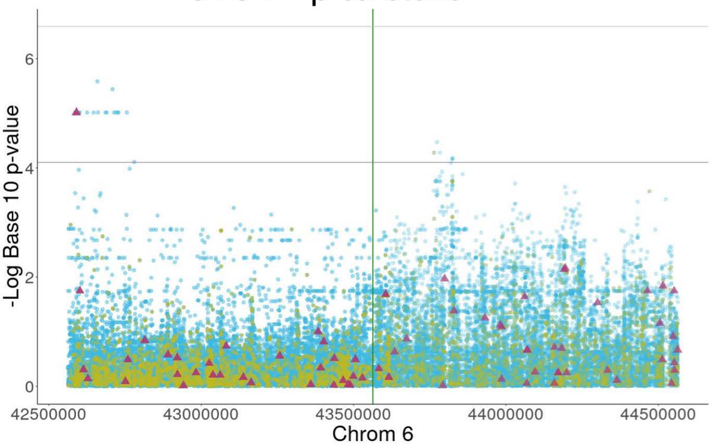

CrtL- ε – lutein

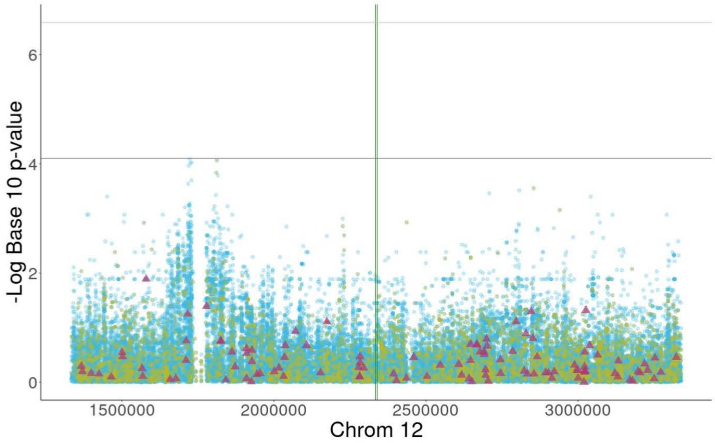

CCD1 – β-ionone

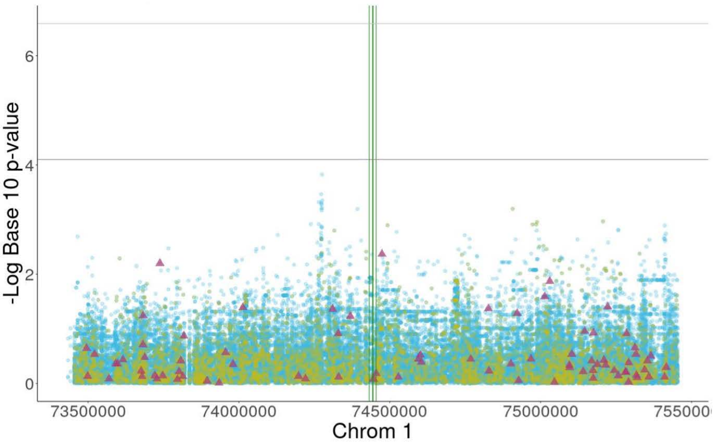

Lipid-derived volatiles

LIP8 – Z-3-hexen-1-ol

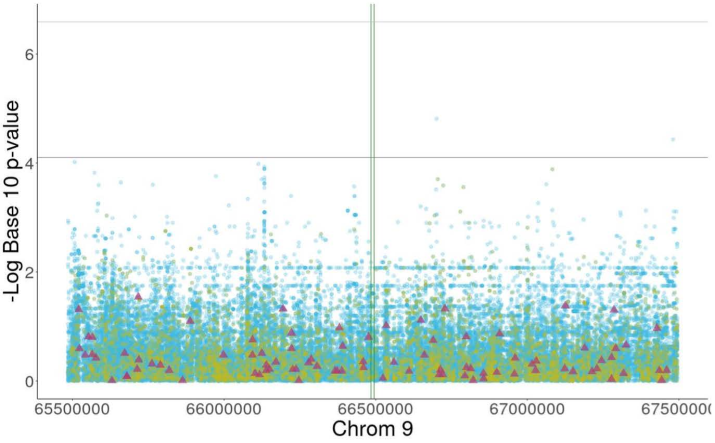

LIP1 – Z-3-hexen-1-ol

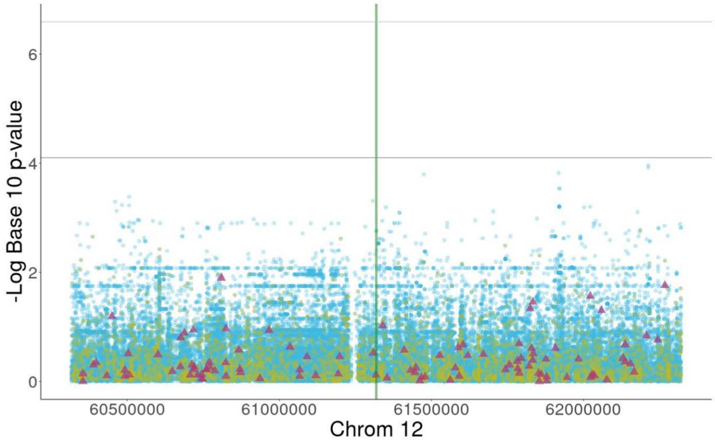

ADH2 – Z-3-hexen-1-ol

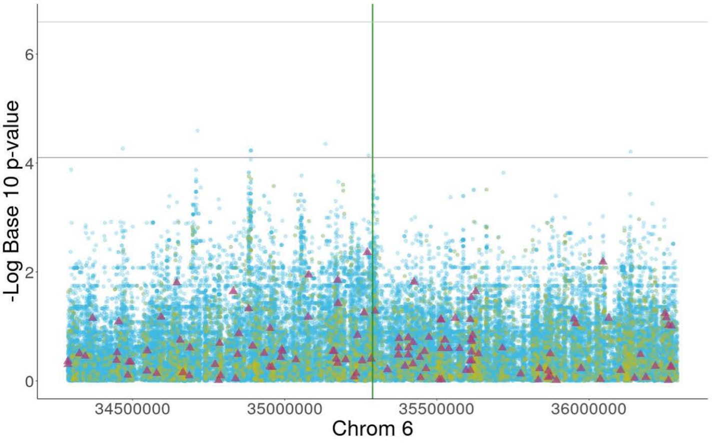

HPL – Z-3-hexen-1-ol

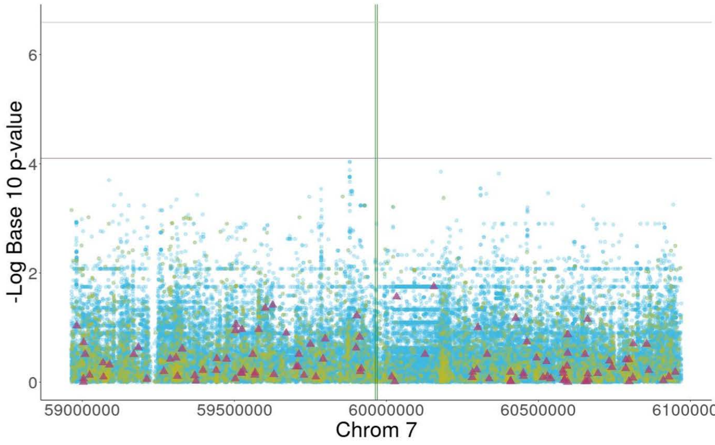

Methylsalicylate and guaiacol

SAMT – methylsalicylate

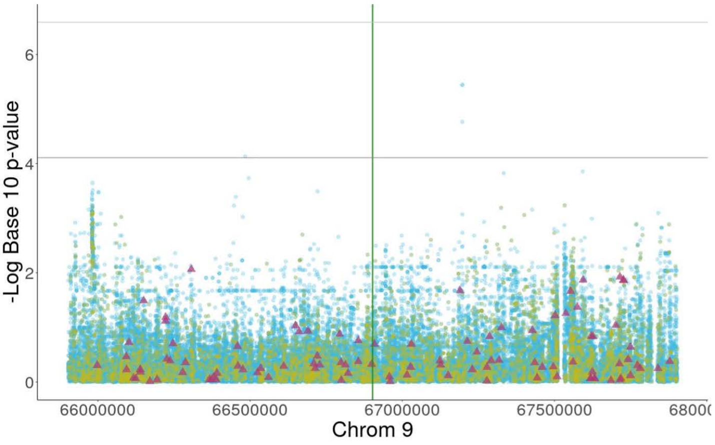

COMT – guaiacol

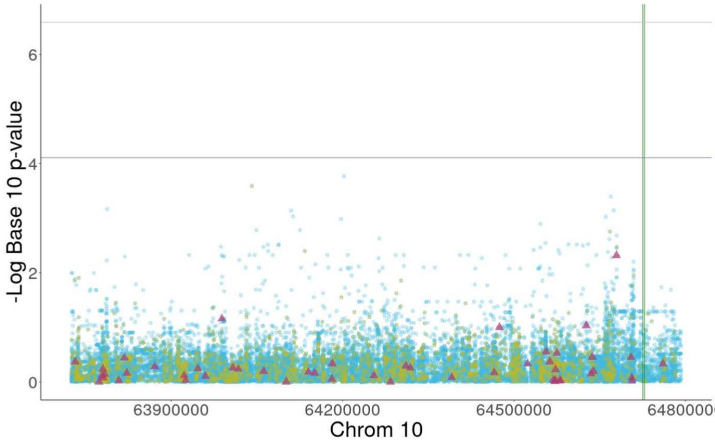

Phenylalanine-derived volatiles

PAR – 2-phenylethanol

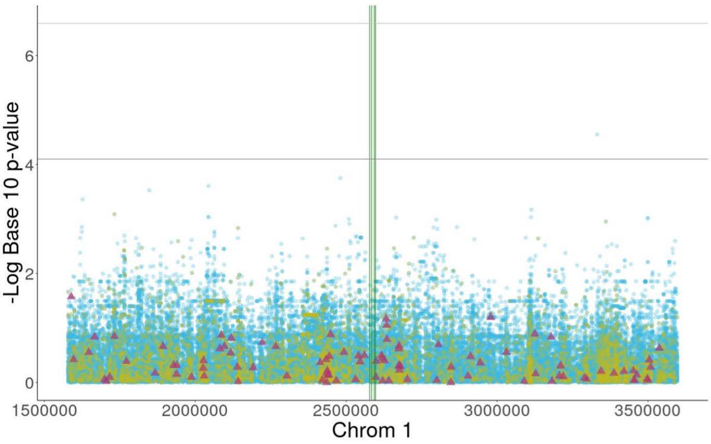

AADC1 – 2-phenylethanol

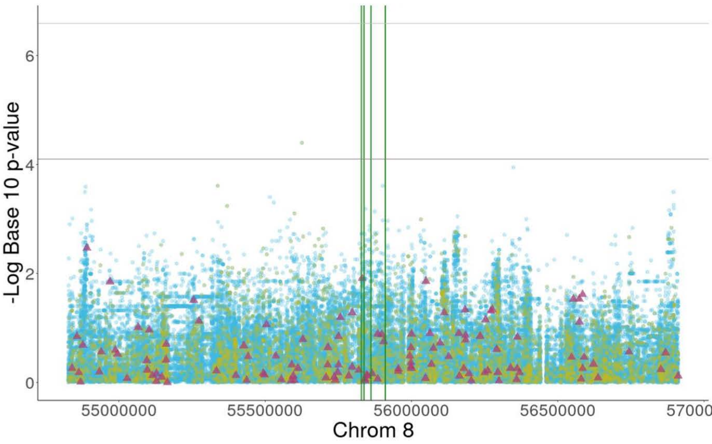

AADC2 – 2-phenylethanol

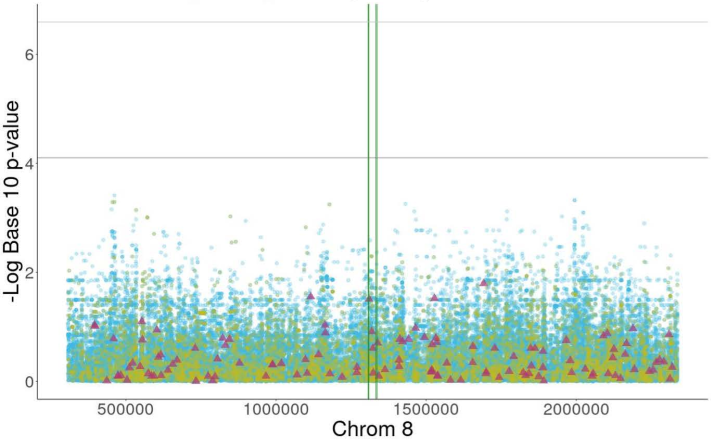

PPEAT – 2-phenylethanol

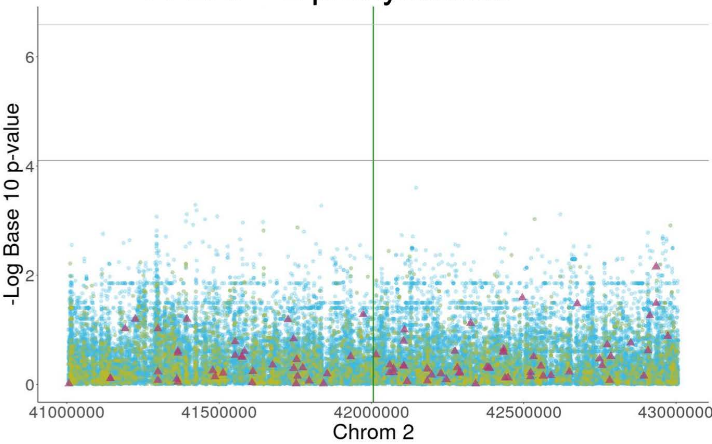

FLORAL4 – 2-phenylethanol

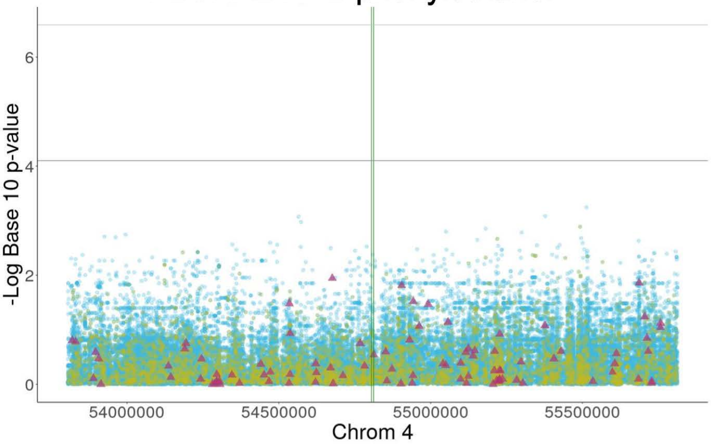

Supplement: Supplementary Figure 1 — Local association mapping for flavor-related genes and their corresponding metabolites. SNPs are plotted as blue dots, INDELs as yellow dots and SVs as purple triangles. Horizontal lines represent 0.05 and 0.01 significance thresholds. [file Image_1.pdf]
