# Supplementary figures and images for: Natural Genetic Diversity in Tomato Flavor Genes
Source: Front Plant Sci. 2021 Jun 4;12:642828. doi: 10.3389/fpls.2021.642828 (PMC8212054; doi:10.3389/fpls.2021.642828)

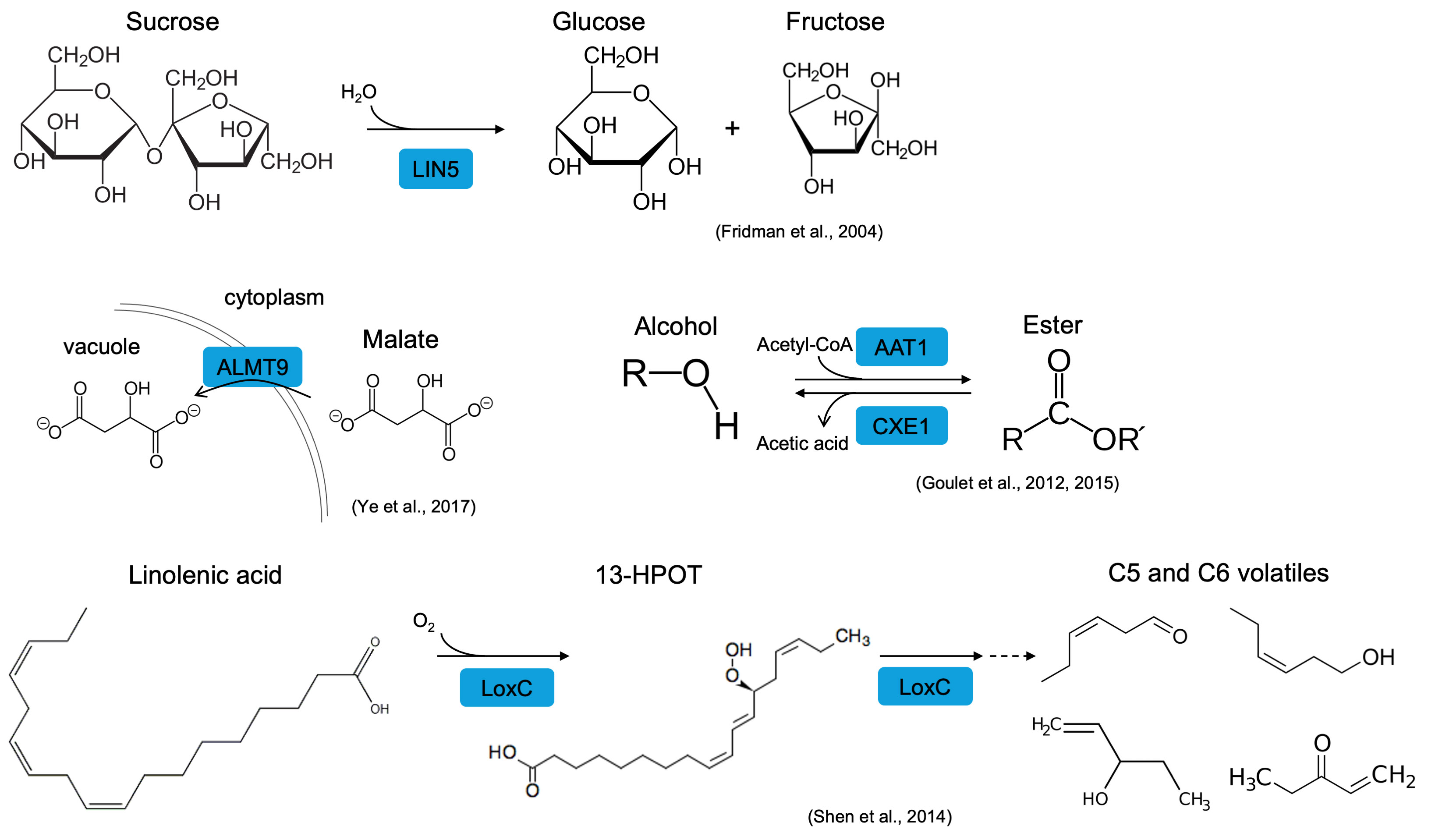

Supplement: Supplementary Figure 2 — Function of the known genes used in this study, as detailed previously in the literature. [file Image_2.jpeg]

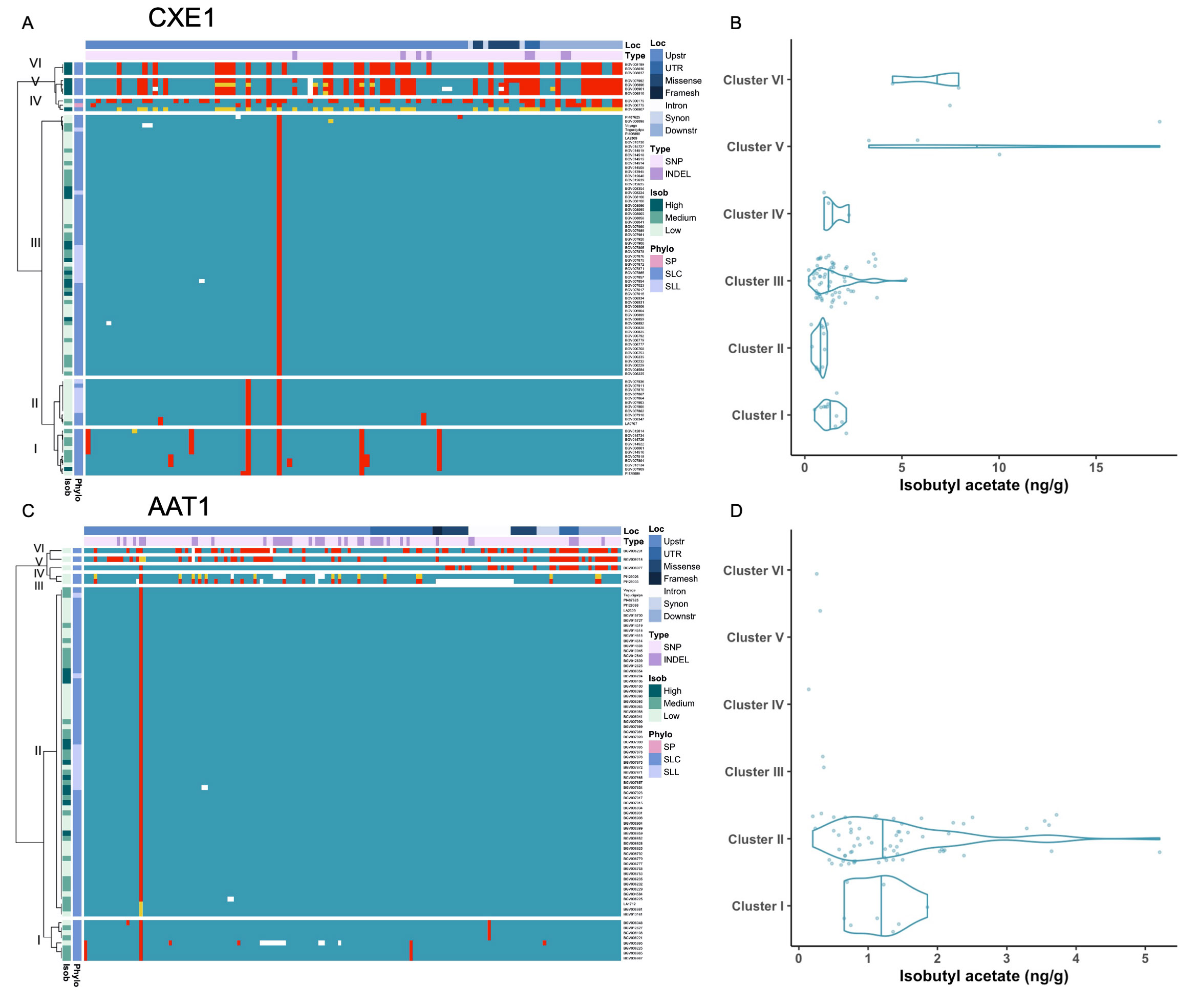

Supplement: Supplementary Figure 4 — Haplotype analysis of AAT1 and CXE1 A. Heatmap of AAT1 including only accessions which belong to Cluster VI in CXE1 clustering B. Violin plots of the isobutyl acetate content classified by haplotype cluster. C. Heatmap of CXE1 including only accessions which belong to Cluster VI in AAT1 clustering D. Violin plots of the isobutyl acetate content classified by haplotype cluster. [file Image_4.jpeg]

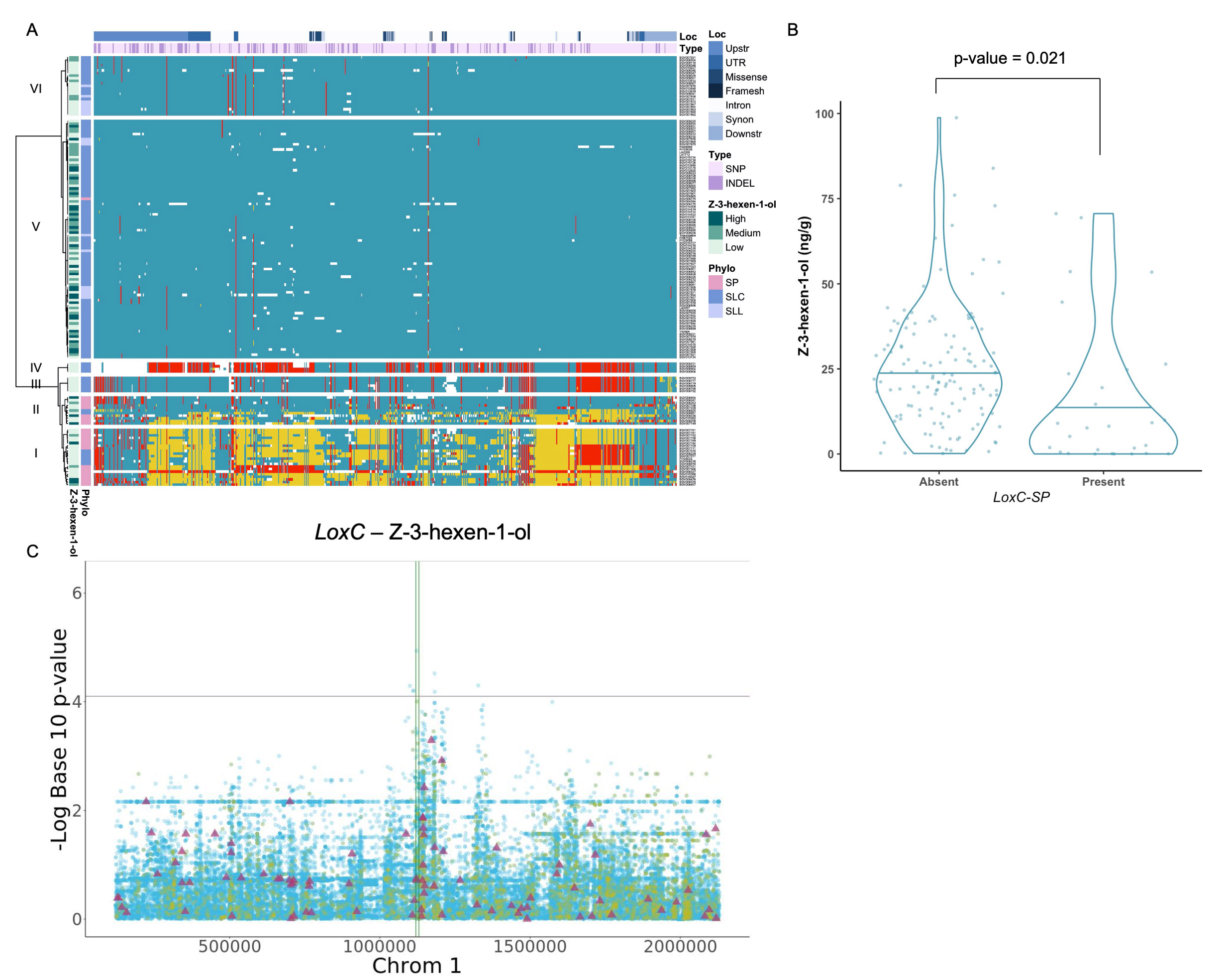

Supplement: Supplementary Figure 5 — Haplotype analysis of LoxC locus for the complete set of accessions. (A) Heatmap representing the genotypes of accessions (rows) for the polymorphisms identified (columns). Reference genotype are represented in blue, alternate in red, heterozygous in yellow and missing data in white. (B) Violin plots of the Z-3-hexen-1-ol content for accessions carrying the duplication (LoxC-SP present) and without the duplication (LoxC-SP absent). [file Image_5.jpeg]

# *LIN5*

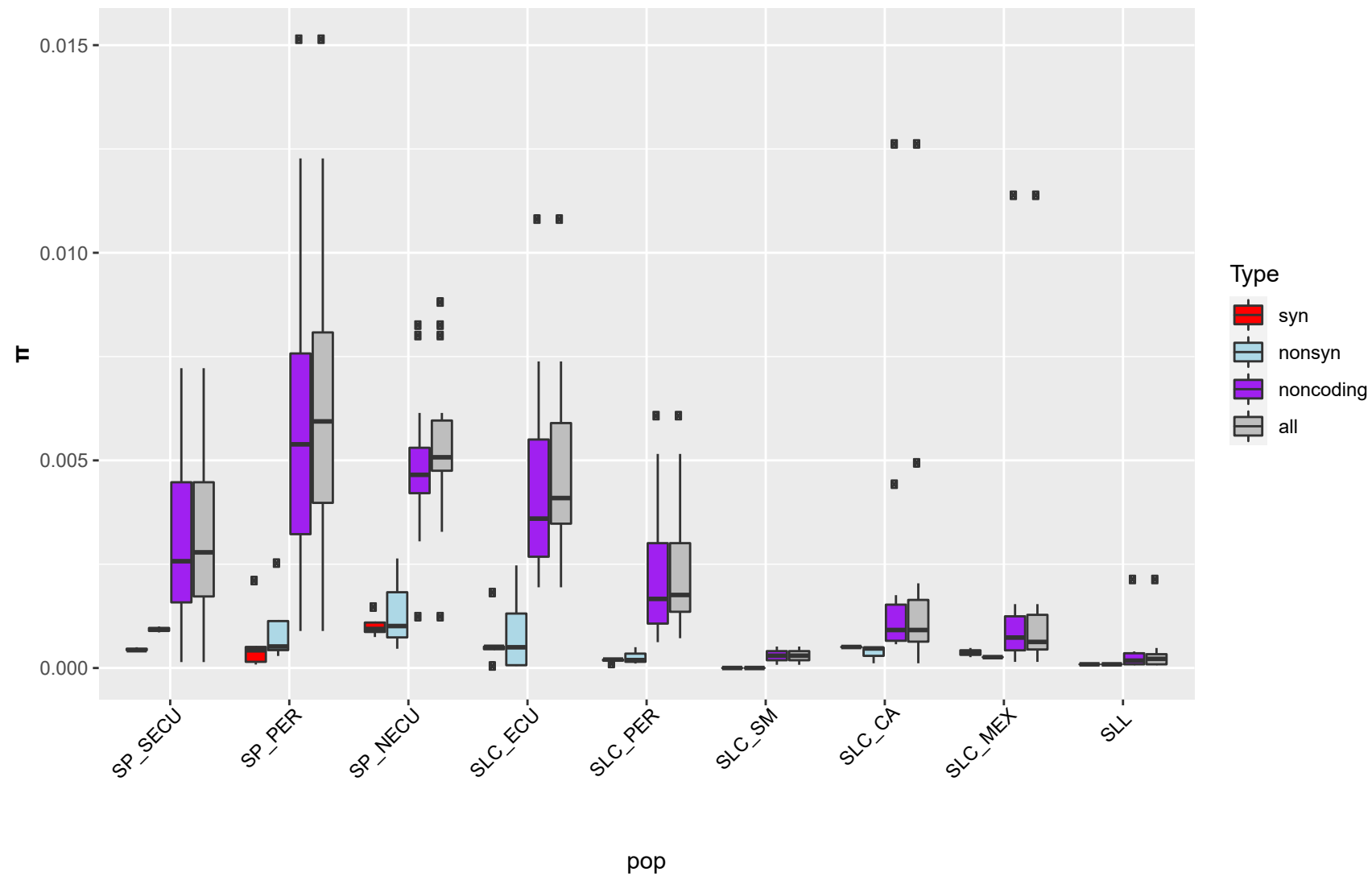

## ALMT9

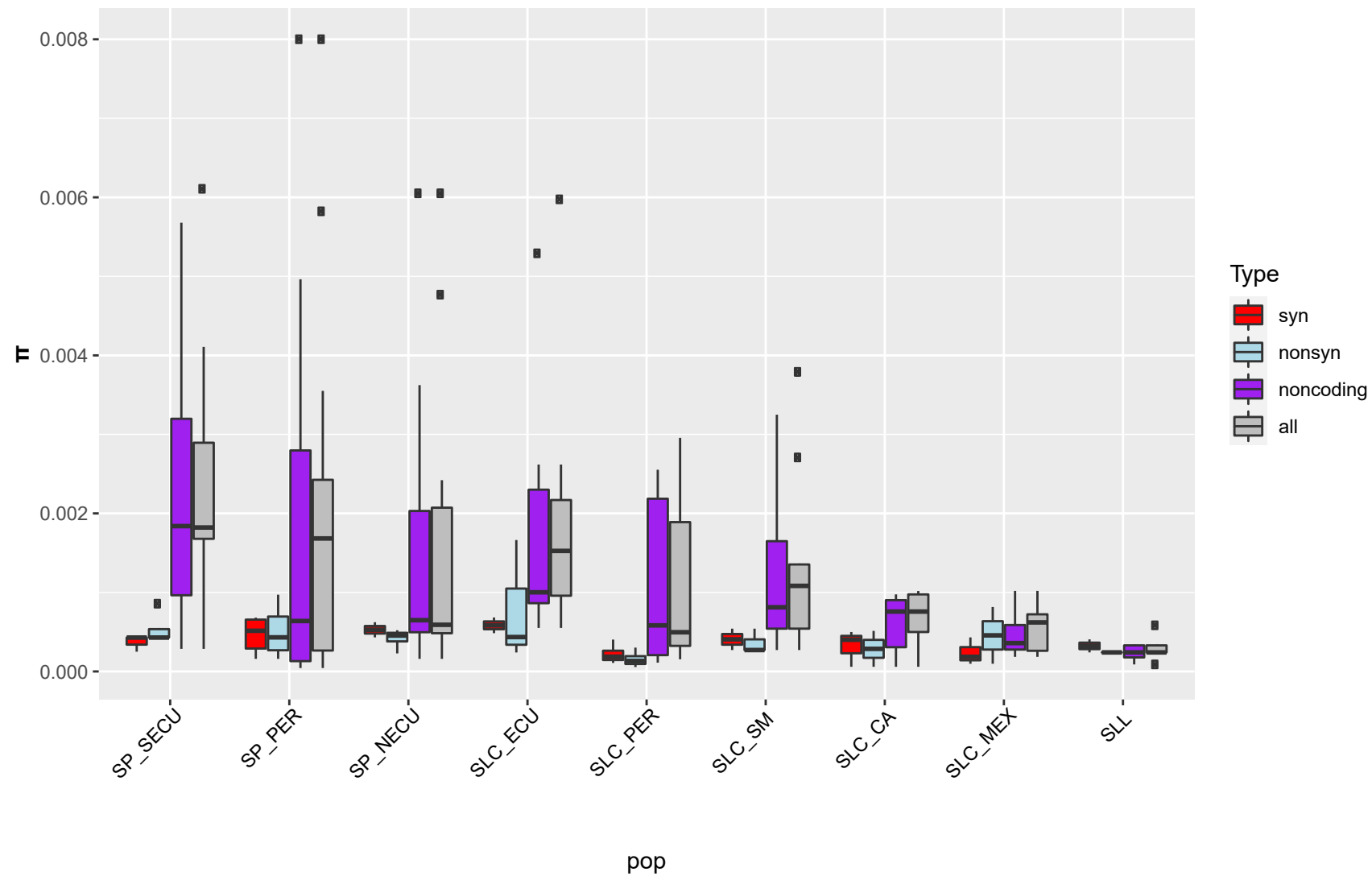

## CXE1

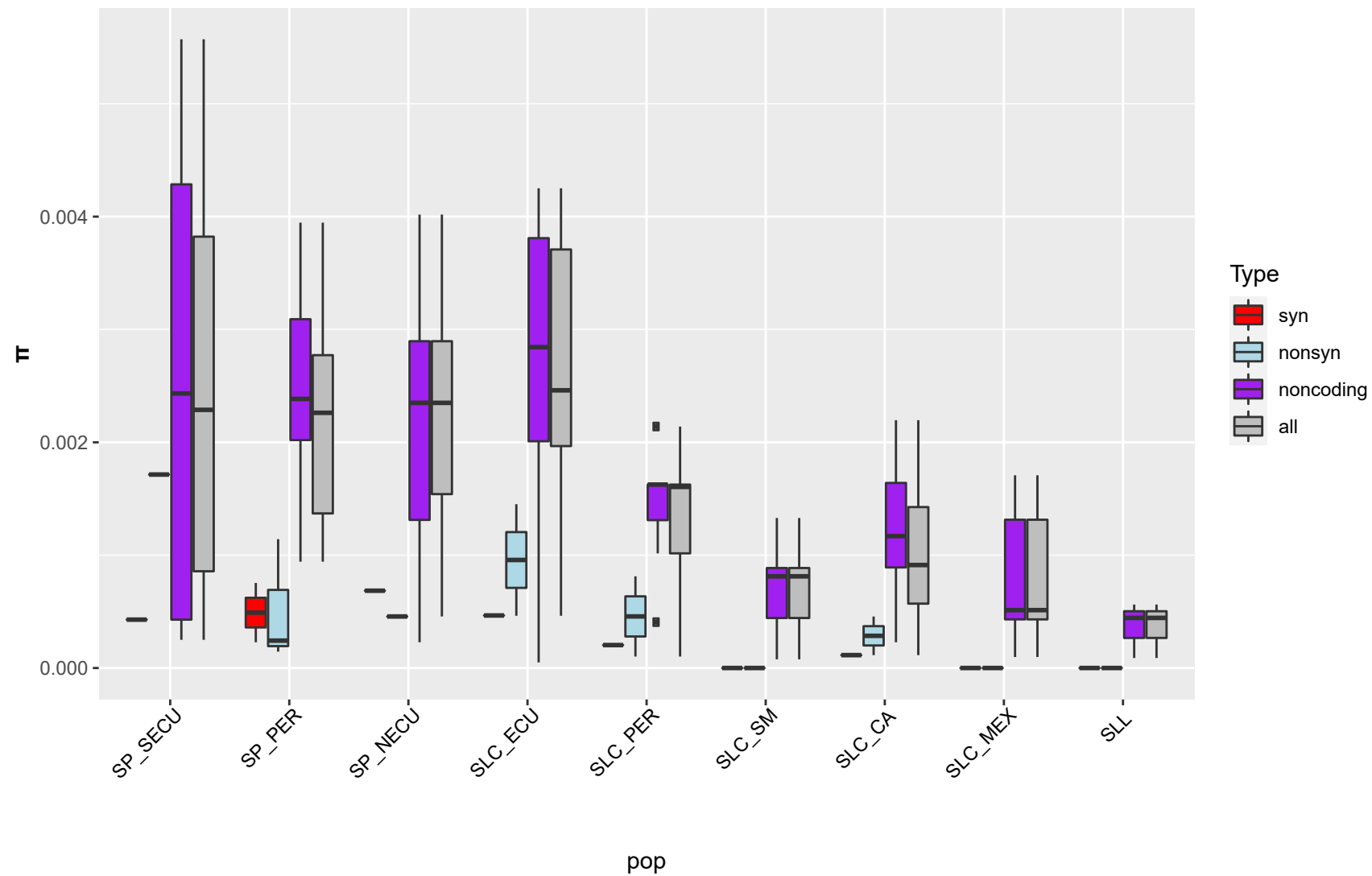

# AAT1

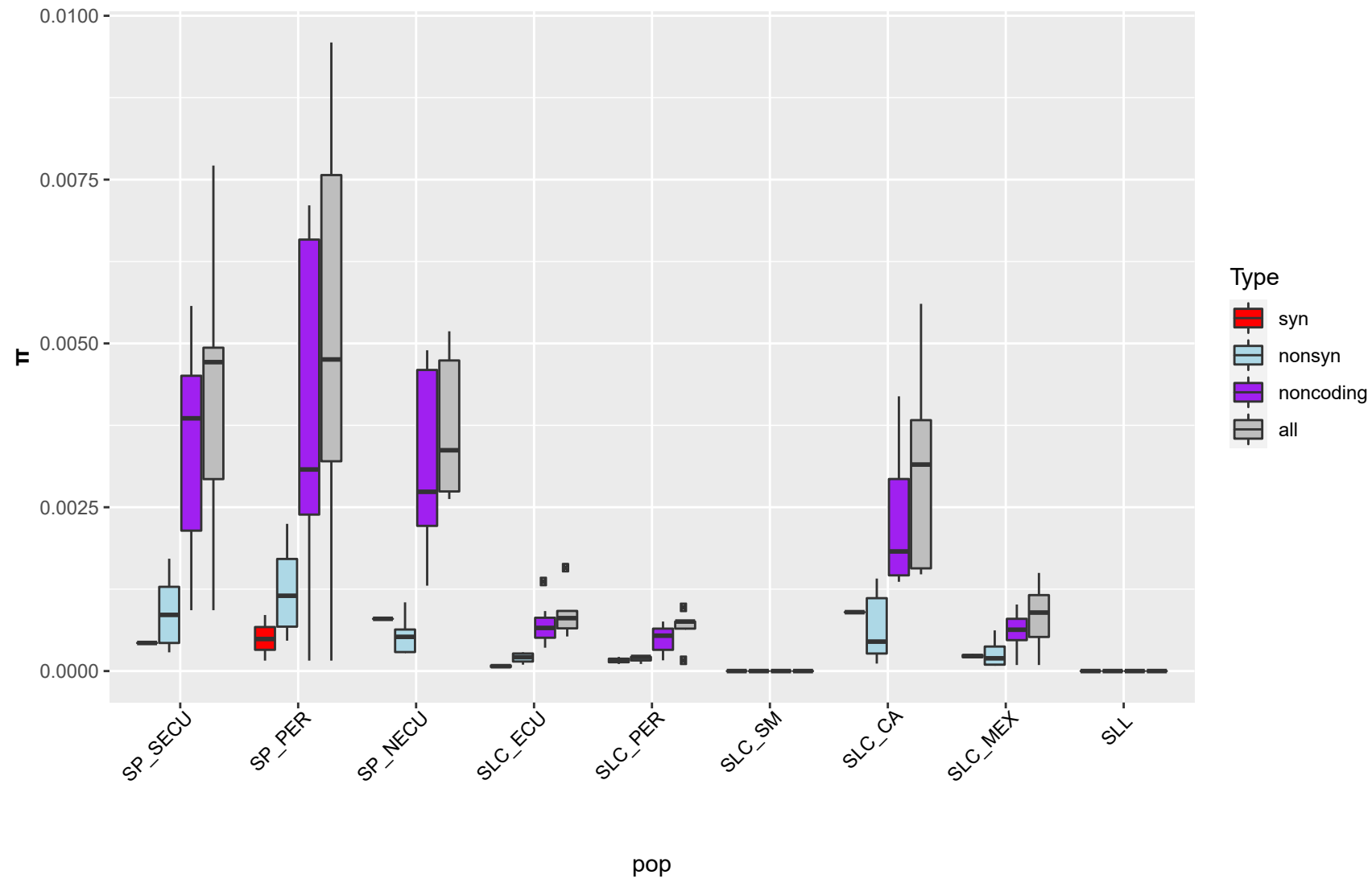

*LoxC*

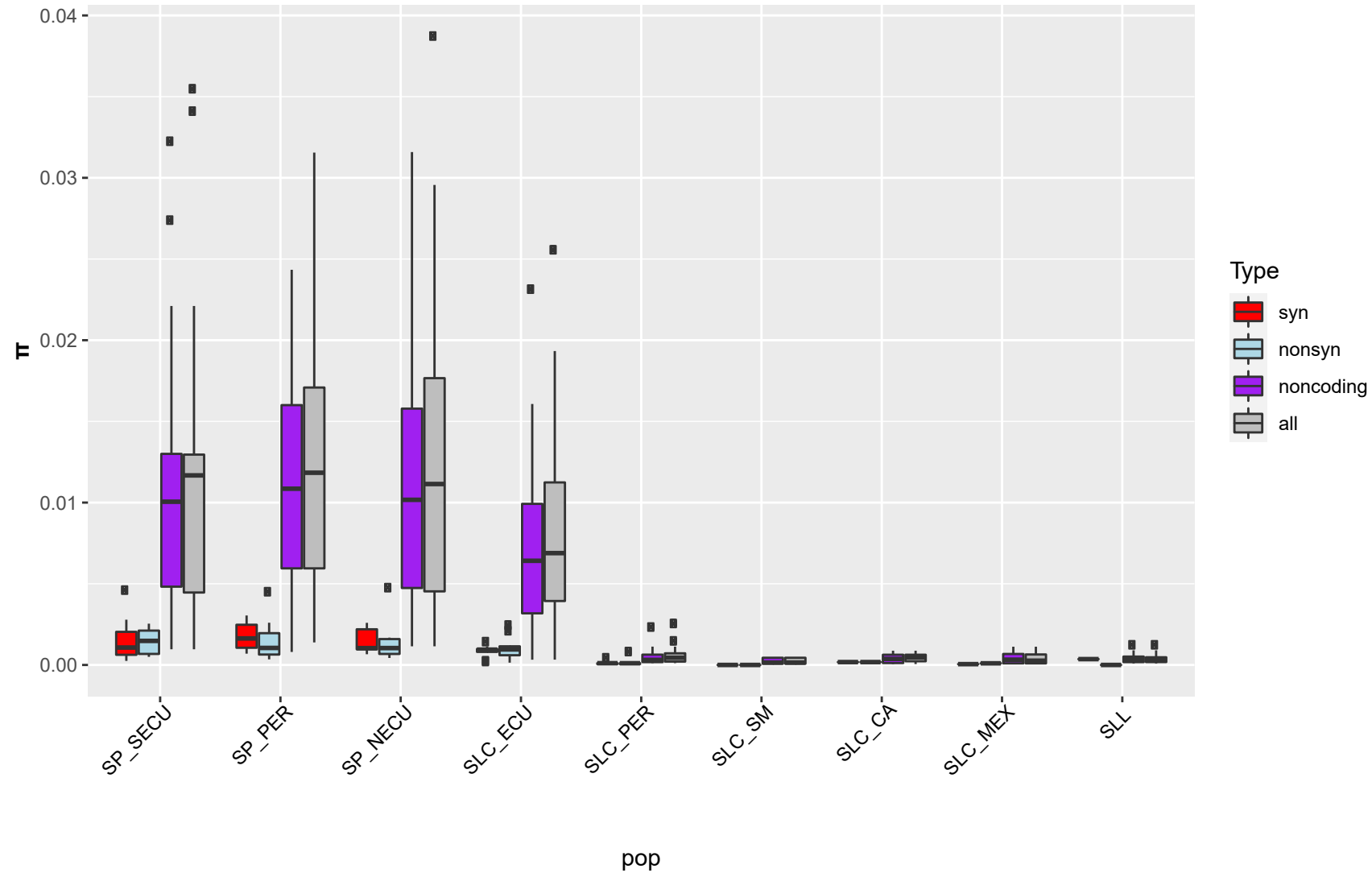

Supplement: Supplementary Figure 6 — Nucleotide diversity in the gene regions, including flanking sequences 3 kb upstream and 1 kb downstream, within each subpopulation. [file Image_6.pdf]

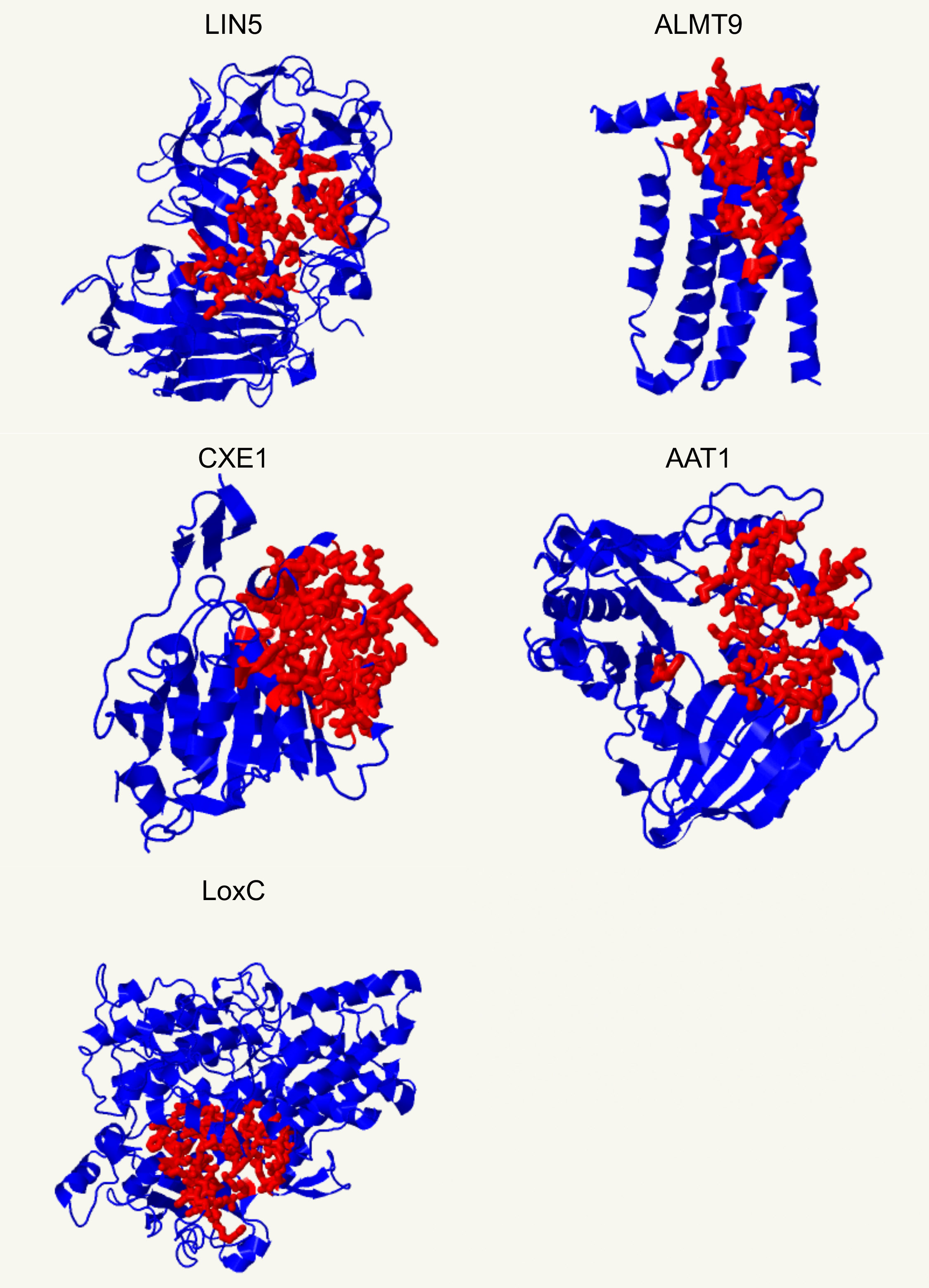

Supplement: Supplementary Figure 7 — Protein modeling predictions of the five proteins using amino acid sequences. The predicted pocket of the enzyme is displayed in red. [file Image_7.jpeg]
