# Supplementary material for: Natural Genetic Diversity in Tomato Flavor Genes
Source: Front Plant Sci. 2021 Jun 4;12:642828. doi: 10.3389/fpls.2021.642828 (PMC8212054; doi:10.3389/fpls.2021.642828)
Supplement: Supplementary Figure 3 — Linkage disequilibrium of SNPs in the gene regions. [file Image_3.pdf]

LIN5

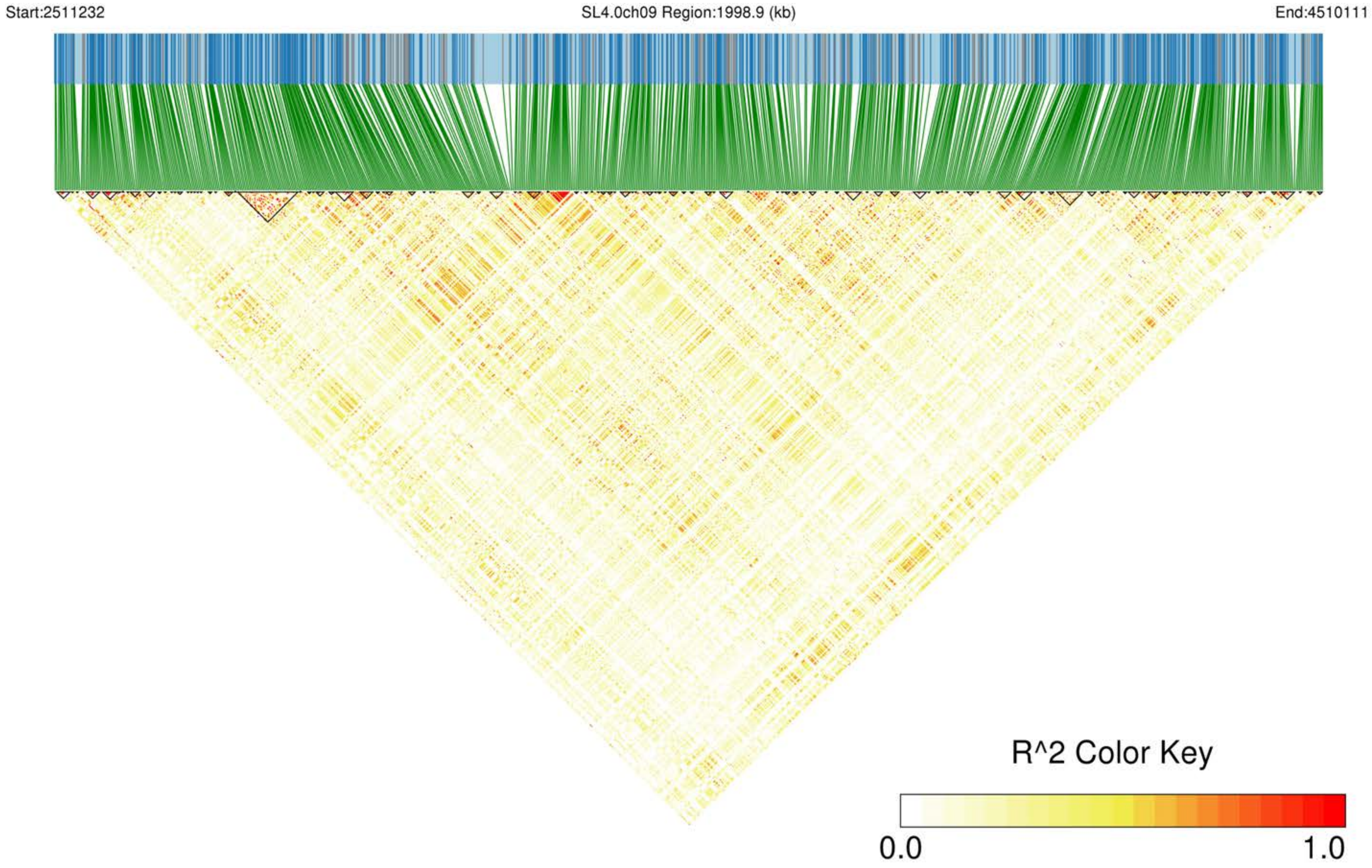
Start:2511232
SL4.0ch09 Region:1998.9 (kb)
End:4510111
R^2 Color Key
0.0
1.0

ALMT9

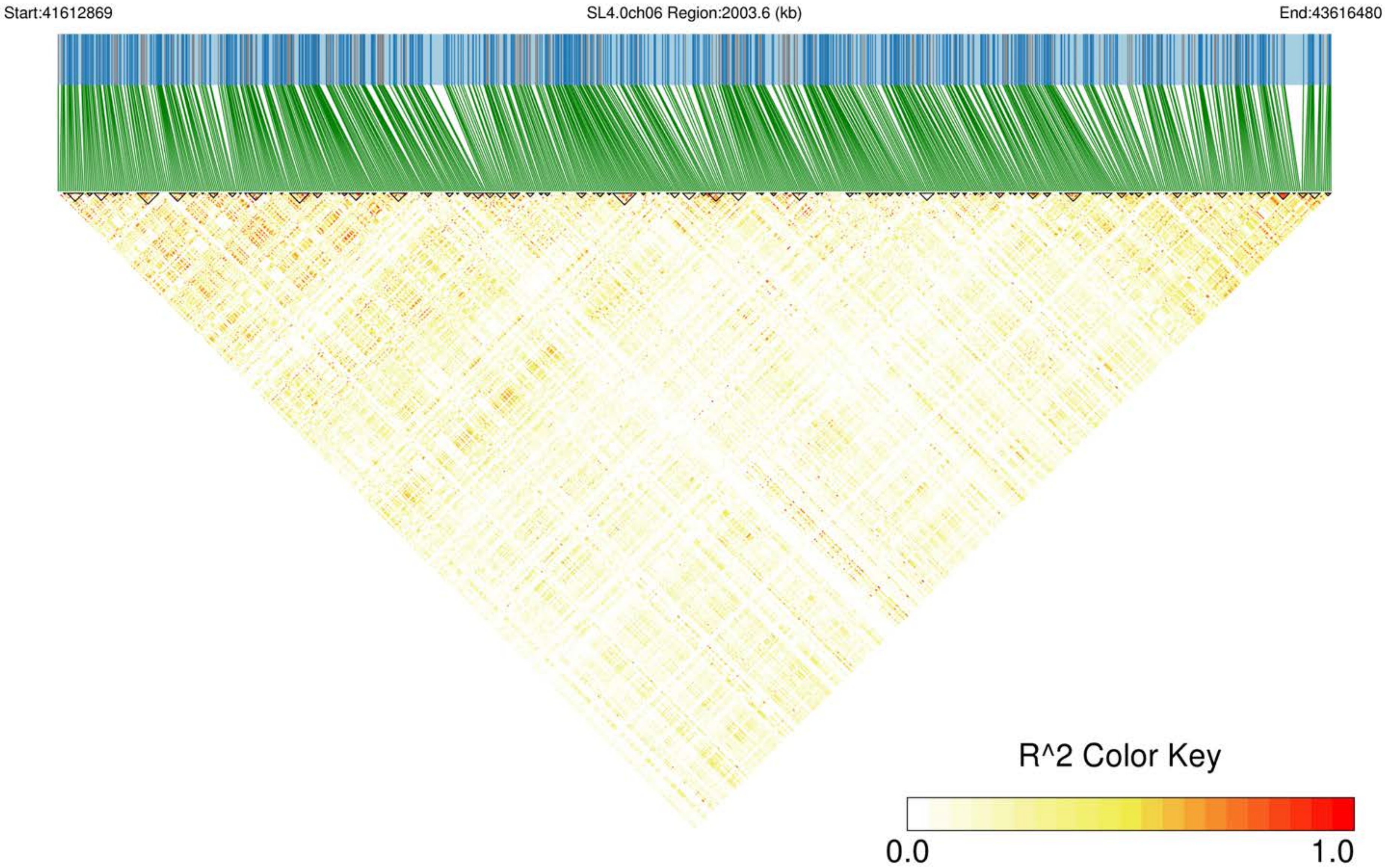

Start:41612869
SL4.0ch06 Region:2003.6 (kb)
End:43616480
R^2 Color Key
0.0
1.0

CXE1

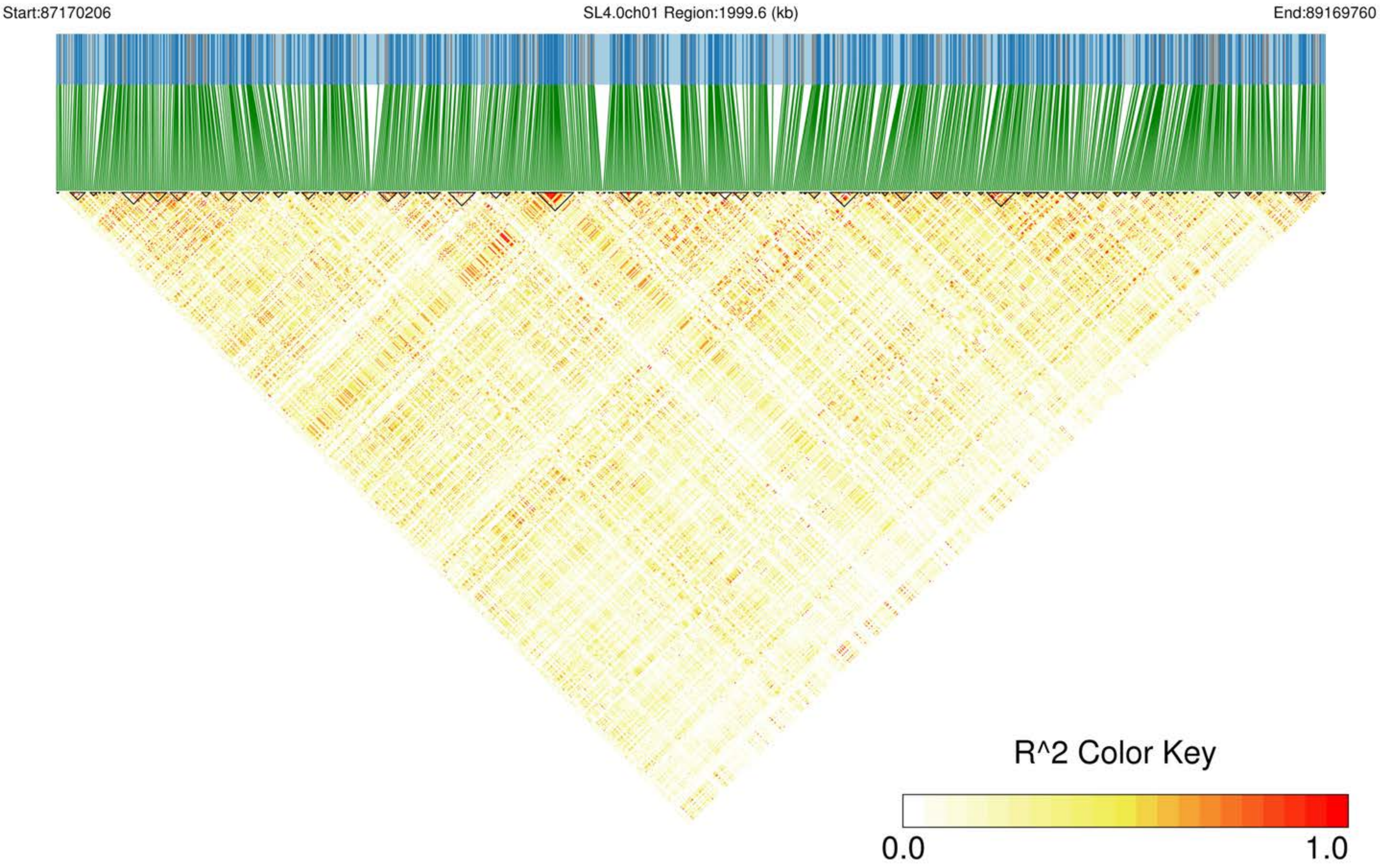
Start:87170206
SL4.0ch01 Region:1999.6 (kb)
End:89169760
R^2 Color Key
0.0
1.0

AAT1

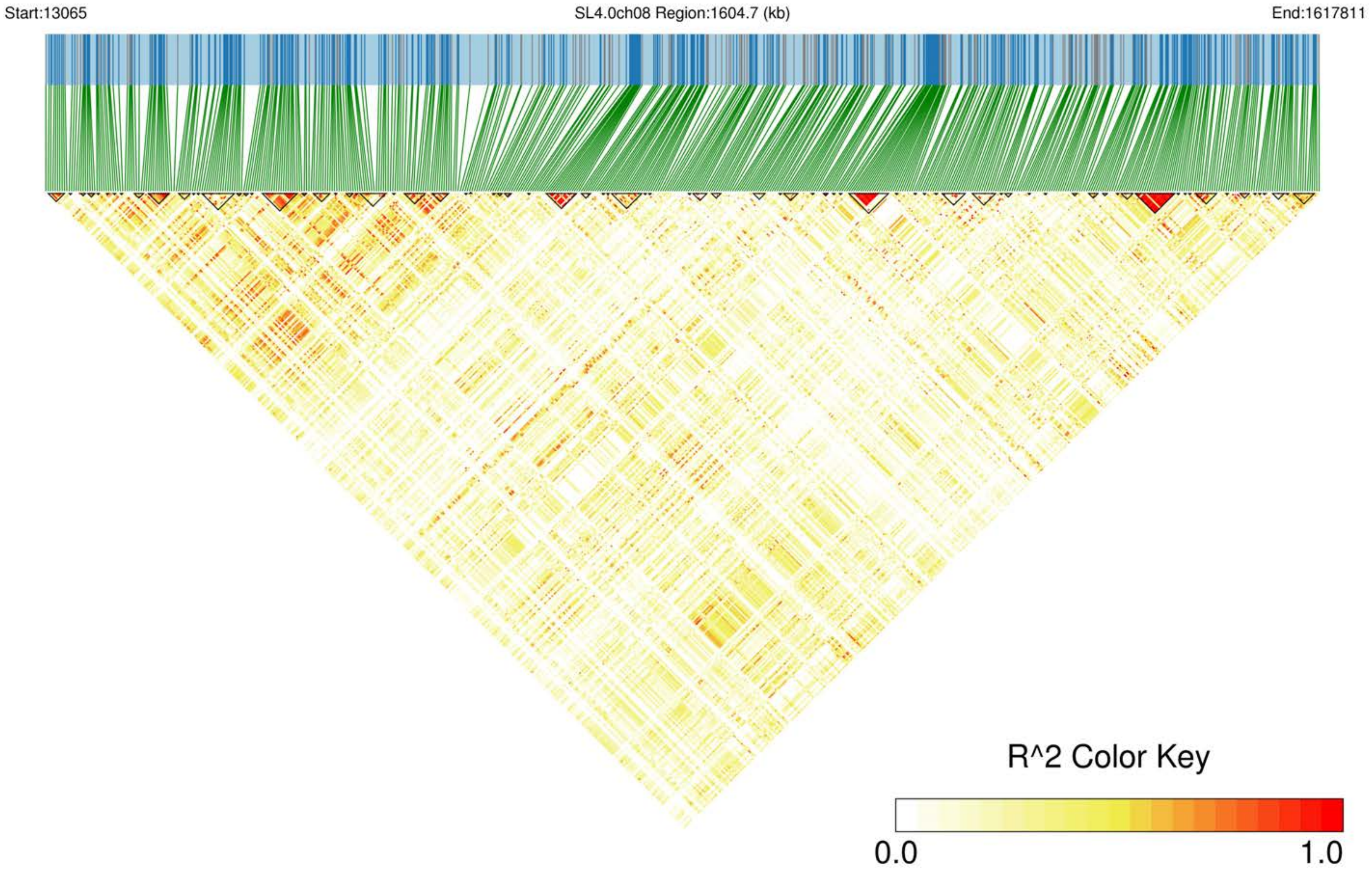

Start:13065
SL4.0ch08 Region:1604.7 (kb)
End:1617811
R^2 Color Key
0.0
1.0

LoxC

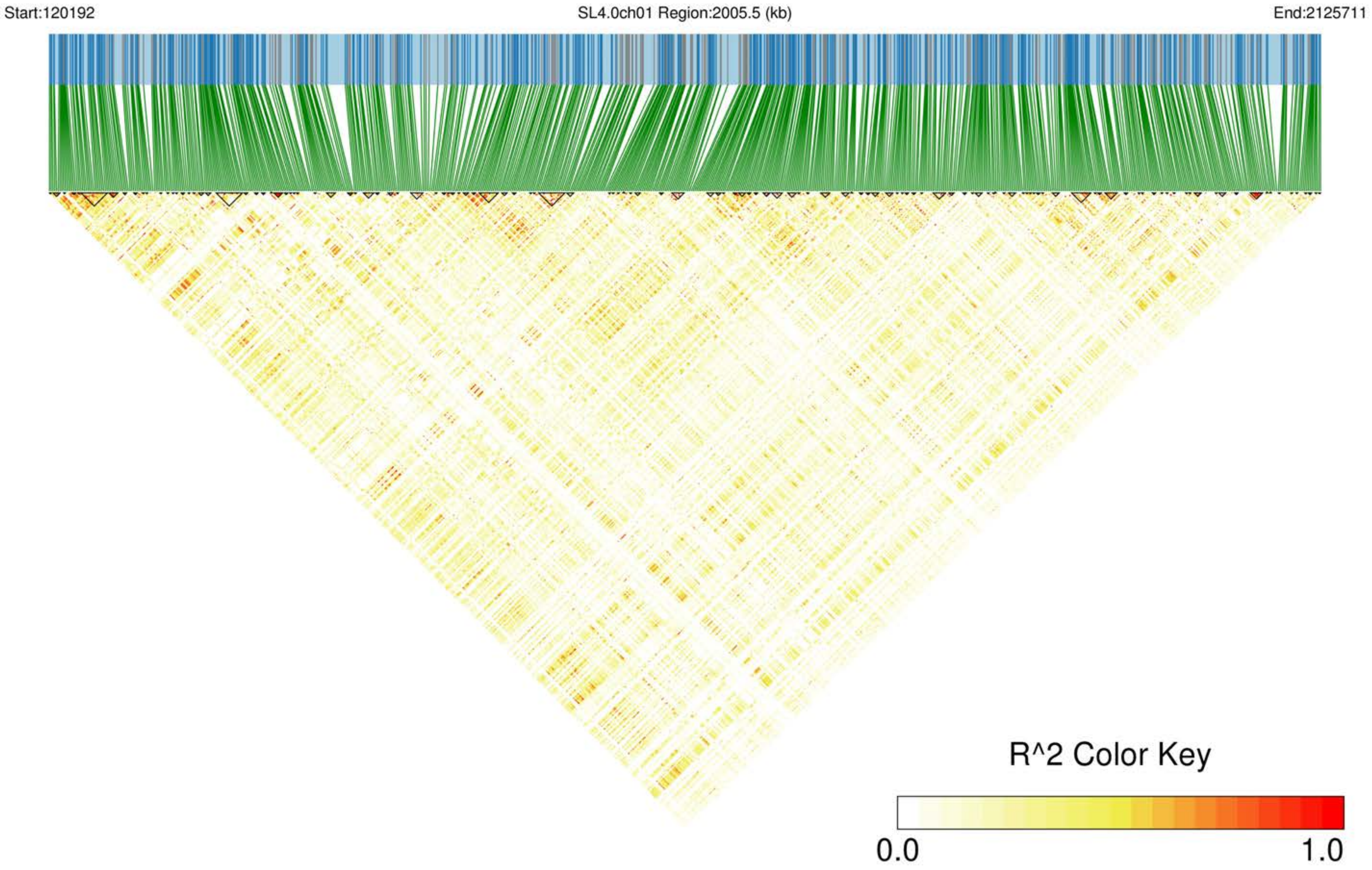

Start:120192
SL4.0ch01 Region:2005.5 (kb)
End:2125711
R^2 Color Key
0.0
1.0
