# Supplementary material for: Natural Genetic Diversity in Tomato Flavor Genes
Source: Front Plant Sci. 2021 Jun 4;12:642828. doi: 10.3389/fpls.2021.642828 (PMC8212054; doi:10.3389/fpls.2021.642828)
Supplement: Supplementary Table 8 — Enzymatic activity of reference and alternate LIN5 alleles. [file Table_8.docx]

Supplementary Table 8. Enzymatic activity of reference and alternate LIN5 alleles.

|  | **Km** | **Vmax** |
| --- | --- | --- |
| LIN5-Asn^366^ | 25.208 | 22.421 |
| LIN5-Asp^366^ | 13.711 | 21.881 |
